# The potential impact of a vaccine on *Neisseria gonorrhoeae* prevalence among heterosexuals living in a high prevalence setting

**DOI:** 10.1016/j.vaccine.2023.07.048

**Published:** 2023-07-28

**Authors:** Thilini N. Padeniya, Ben B. Hui, James G. Wood, Kate L. Seib, David G. Regan

**Affiliations:** a Institute for Glycomics, Griffith University, Gold Coast, Queensland, Australia; b The Kirby Institute, University of New South Wales, Sydney, New South Wales, Australia; c School of Population Health, University of New South Wales, Sydney, New South Wales, Australia

**Keywords:** *Neisseria gonorrhoeae*, Gonorrhoea, Sexually transmitted infection, Gonorrhoea vaccine, Mathematical model, Compartmental model

## Abstract

**Background:**

Treatment of *Neisseria gonorrhoeae* is under threat with the emergence and spread of antimicrobial resistance. Thus, there is a growing interest in the development of a gonorrhoea vaccine. We used mathematical modelling to assess the impact of a hypothetical vaccine in controlling gonorrhoea among heterosexuals living in a setting of relatively high *N. gonorrhoeae* prevalence (~3 %).

**Methods:**

We developed a mathematical model of *N. gonorrhoeae* transmission among 15–49-year-old heterosexuals, stratified by age and sex, and calibrated to prevalence and sexual behaviour data from South Africa as an example of a high prevalence setting for which we have data available. Using this model, we assessed the potential impact of a vaccine on *N. gonorrhoeae* prevalence in the entire population. We considered gonorrhoea vaccines having differing impacts on *N. gonorrhoeae* infection and transmission and offered to different age-groups.

**Results:**

The model predicts that *N. gonorrhoeae* prevalence can be reduced by ~50 % in 10 years following introduction of a vaccine if annual vaccination uptake is 10 %, vaccine efficacy against acquisition of infection is 25 % and duration of protection is 5 years, with vaccination available to the entire population of 15–49-year-olds. If only 15–24-year-olds are vaccinated, the predicted reduction in prevalence in the entire population is 25 % with equivalent vaccine characteristics and uptake. Although predicted reductions in prevalence for vaccination programmes targeting only high-activity individuals and the entire population are similar over the same period, vaccinating only high-activity individuals is more efficient as the cumulative number of vaccinations needed to reduce prevalence in the entire population by 50 % is ~3 times lower for this programme.

**Conclusion:**

Provision of a gonorrhoea vaccine could lead to substantial reductions in *N. gonorrhoeae* prevalence in a high prevalence heterosexual setting, even with moderate annual vaccination uptake of a vaccine with partial efficacy.

## 1. Introduction

Gonorrhoea is a sexually transmitted infection (STI) caused by the bacterium *Neisseria gonorrhoeae. N. gonorrhoeae* infects the urogenital epithelia, in particular the urethra in men and cervix in women, as well as the rectum, oropharynx and sometimes the eyes in both men and women [1]. If untreated, serious complications of gonorrhoea, such as pelvic inflammatory disease (PID) in women and epididymitis in men, can occur and can cause infertility [2].

Gonorrhoea remains a major public health concern with infection rates increasing worldwide, and the emergence and spread of antimicrobial resistance (AMR) reducing the effectiveness of most of the antibiotic treatments currently available [3-5]. The World Health Organization (WHO) estimated global prevalence of *N. gonorrhoeae* in 2020 to be 0.9 % in women and 0.7 % in men, aged 15–49 years, and 82.4 million people were newly infected with *N. gonorrhoeae* in 2020 [6]. The WHO African Region had the highest estimated prevalence for gonorrhoea in 2020 at 1.7 % (95 % UI 1.1–2.2 %) and 1.2 % (95 % UI 0.7–2.0 %) for women and men, respectively [6]. *N. gonorrhoeae* AMR is considered a serious global threat by the WHO [7] which, along with other organizations such as the National Institute of Allergy and Infectious Diseases (NIAID), has identified the development of a gonorrhea vaccine as an urgent priority [8-10].

Observational studies have detected a decrease in incidence of gonorrhoea following vaccination with the *Neisseria meningitidis* serogroup B outer membrane vesicle (OMV)-based vaccines MeNZB [11] and 4CMenB [12,13], with a predicted vaccine efficacy of approximately 30–40 % against *N. gonorrhoeae* infection. The 4CMenB vaccine induces antibodies that cross-react with *N. gonorrhoeae*, further supporting the potential protection against gonorrhoea [14]. These findings have provided additional momentum for gonorrhoea vaccine development. Several mathematical modelling studies have assessed the potential impact of a gonorrhoea vaccine in controlling gonorrhoea, and suggest that a gonorrhoea vaccine of moderate efficacy could rapidly and substantially reduce *N. gonorrhoeae* prevalence in high-risk populations of men who have sex with men (MSM) [15-17], as well as in heterosexual populations having low to moderate *N. gonorrhoeae* prevalence (~0.1–2 %) [18-20]. However, previous modelling studies have not focused on low- and middle-income countries (LMIC) settings, nor have they assessed the impact of a gonorrhoea vaccine in a high-risk heterosexual population setting with relatively high prevalence or considered how age-group targeted vaccinations programmes and vaccines with different mechanism of protection may impact prevalence. In this study, we use a mathematical model, calibrated to replicate the sexual behaviour observed in a heterosexual population living in a district of KwaZulu-Natal, South Africa where the prevalence of *N. gonorrhoeae* is 2.8 % among 15–49-year-olds [21] to estimate the potential impact on population prevalence. We consider vaccination for different age-groups and evaluate different scenarios regarding the mechanism of action of hypothetical gonorrhoea vaccines.

## 2. Methods

**Fig. 1.**
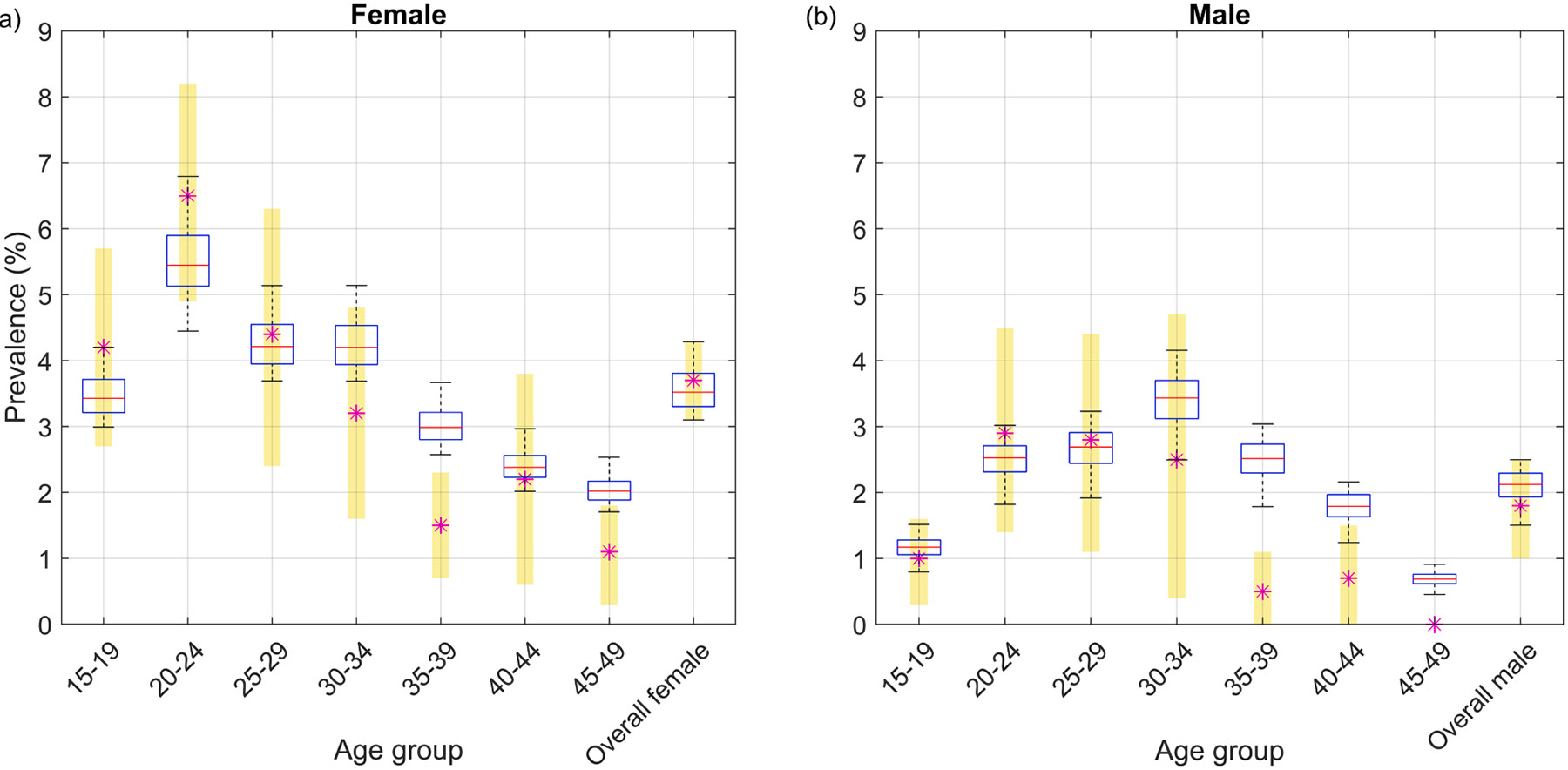
Reported versus model-predicted *N. gonorrhoeae* prevalence for females and males. The reported median prevalence is indicated by an asterisk (*) with the 95 % confidence intervals denoted by yellow rectangles. The model predicted prevalence for the 572 parameter sets that produced outcomes comparable to the reported overall female and male prevalence are shown using a box and whisker representation. In each box, the red horizontal line indicates the median, bottom and top edges of the box indicate the 25th and 75th percentiles, respectively, and whiskers indicate the maximum and minimum values for the model-predicted age-group specific prevalence.

We developed a Susceptible-Infected-Recovered-Susceptible (SIRS) type compartmental model, to simulate the transmission of *N. gonorrhoeae* among 15–49-year-olds living in a high prevalence setting, similar to that observed in a South African setting [22]. The model accounts for symptomatic and asymptomatic infection, *N. gonorrhoeae* testing and treatment, and the modelled population is stratified by age and sexual activity. The model is calibrated to age-specific female and male *N. gonorrhoeae* prevalence data from a district in KwaZulu-Natal, South Africa for the year 2015 [21]. We consider the South African setting KwaZulu-Natal in our model because it is a high prevalence setting for which *N. gonorrhoeae* prevalence data was available for calibration. However, this context should be considered as illustrative, with our focus being high prevalence settings in general. Calibration is achieved by adjusting the average partner acquisition rate for the entire population, the proportion of acts in which condoms are used in sexual partnerships, and the average number of sexual acts per week (keeping all the other parameters fixed), such that a stable *N. gonorrhoeae* prevalence matching the target prevalence is established prior to the introduction of vaccination (see Table S2 and Section 3 of the Supplementary material). Then, to account for uncertainty in real-world *N. gonorrhoeae* prevalence, natural history parameters, genderspecific per-act transmission probabilities and proportions of infections that are symptomatic were varied (see Table S6 for ranges used), thereby producing a range of model outcomes. Latin hypercube sampling (LHS) was used to generate 10,000 random samples for these parameters and the model was run for these 10,000 parameters sets until dynamic equilibrium was reached. Then, at equilibrium, we identified 572 parameter sets that produced overall female and male *N. gonorrhoeae* prevalence comparable to reported data. In Fig. 1, we present the reported and the model-predicted age-group-specific and overall *N. gonorrhoeae* prevalence for females and males. A full description of model implementation, parameter derivations and ranges, and calibration method is provided in the Supplementary Material.

The model with vaccination incorporated is illustrated schematically in Fig. S1 and described in detail in the Supplementary Material. Vaccination scenarios are summarised in Table 1. Briefly, in our implementation of vaccination, unvaccinated individuals are moved to the vaccinated state and vaccine-conferred protection then wanes over time whereupon vaccinated individuals move to the vaccination waned state. The proportion of eligible unvaccinated individuals that are vaccinated in a year is referred to henceforth as the “annual vaccination uptake” and we vary this under each vaccination scenario (Table 1). In South Africa, 45 % and 58 % of men and women, respectively, are tested for HIV annually [23]. In order to arrive at a feasible level of vaccination coverage in this study we assumed that 10 %, 20 %, 40 % or 80 % of the proportion who would annually be tested for HIV may receive vaccination against *N. gonorrhoeae* infection. Based on the product of these proportions, we assumed for simplicity that annual vaccination uptake for both men and women was 5 %, 10 %, 20 % or 40 %, with uniform likelihood of receiving vaccine across the targeted populations. If in reality uptake of vaccination was limited to a subgroup of the population and/or a proportion of the population are never vaccinated, our findings are likely to over-estimate the impact of vaccination. To assess the impact of vaccinating different age-groups, we consider the impact of providing vaccination to; 1) the entire population (defined as the sexually active population aged 15–49 years); 2) 15–24-year-olds only; or 3) 15–19-year-olds only. For a subset of the cases listed under scenario 1 in Table 1, we also considered an additional vaccination scenario whereby vaccination is only available to high-activity individuals, defined as those with a sexual partner acquisition rate of ~5 per annum versus ~1 per annum in the low-activity group.

There is currently no vaccine available specifically for the prevention of gonorrhoea, and the properties of gonorrhoea vaccines investigated in our model are therefore hypothetical. Three types of vaccine efficacy were considered (see Supplementary Material for more detail):

1. *Efficacy against acquisition of infection (protective efficacy):* the vaccine reduces susceptibility to infection in vaccinated individuals. For example, 50 % protective efficacy means that all vaccinated people have a 50 % reduced chance of acquiring *N. gonorrhoeae* per sexual contact with an infected partner.
2. *Efficacy against transmission (transmission suppression efficacy):* the vaccine reduces the infectiousness of vaccinated individuals who become infected (i.e., have a breakthrough infection). Here we assume that transmission may be reduced by vaccination due to factors such as reduced bacterial load and/or duration of infection.
3. *Efficacy against symptoms (symptom suppression efficacy):* the vaccine reduces the probability that individuals with breakthrough infection will develop symptoms. Here we assume that while the presence/absence of symptoms does not directly impact transmission rates, treatment will not be actively sought by asymptomatic individuals. We modelled symptom suppression efficacy as some vaccines suppress symptoms but do not always completely prevent infection (e.g., COVID-19 [24] and pertussis [25] vaccines). Here, we assume that while the presence/absence of symptoms does not directly impact transmission rates, treatment will not be actively sought by asymptomatic individuals.

We assessed the impact on *N. gonorrhoeae* prevalence in the entire population for 30 years from 2015 onwards, under the four vaccine scenarios listed in Table 1, with the afore-mentioned age-group-specific vaccination programmes, and for different durations of protection, different types and levels of vaccine efficacy and annual vaccination uptakes.

## 3. Results

**Fig. 2.**
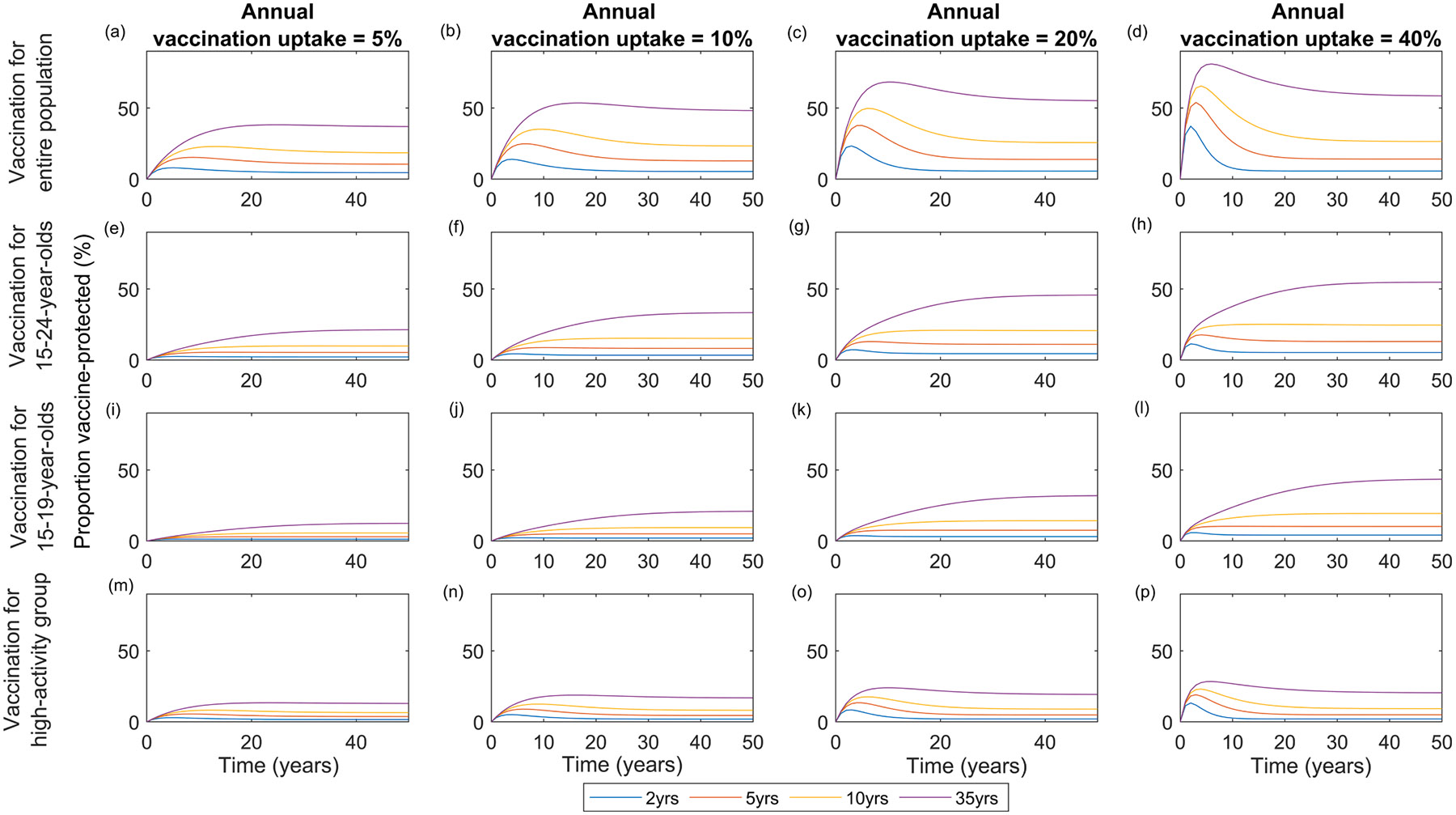
Change in the percentage of people who are vaccine-protected in the population over time for different vaccination programmes, durations of protection and annual vaccination uptakes. Here, the assumed vaccination efficacy against acquisition of infection is 50 %.

In the sections below, we present the results for a range of vaccination scenarios (summarised in Table 1) in terms of their impact over time on *N. gonorrhoeae* prevalence for the entire population. In the figures below, we present the median prevalence for the 572 simulations conducted. The interquartile ranges (IQR) of prevalence for each scenario are presented in Tables S7-S13 of the Supplementary material. Considering scenarios with 5–40 % annual vaccination uptake, the proportion of people who are vaccine-protected (i.e., those who have received the vaccine and protection has not waned yet) reaches a plateau after about 30 to 40 years and varies between 1 % and 60 % depending mainly on the assumed annual vaccination uptake, duration of protection and the target group as shown in Fig. 2, for the entire population, 15–24-, 15–19-year-old or high-activity group vaccination programmes. For instance, for an annual vaccination uptake of 40 %, duration of protection of 10 years, and protective efficacy of 50 %, the proportion of the population that is vaccine-protected is ~50 %, ~30 % or ~20 % after 10 years if vaccination is provided to the entire population, 15–24-year-olds, or 15–19-year-olds, respectively.

### 3.1. Impact of vaccines that alter susceptibility to infection, with 0–100 % protective efficacy

#### a) Age-group-specific vaccination

**Fig. 3.**
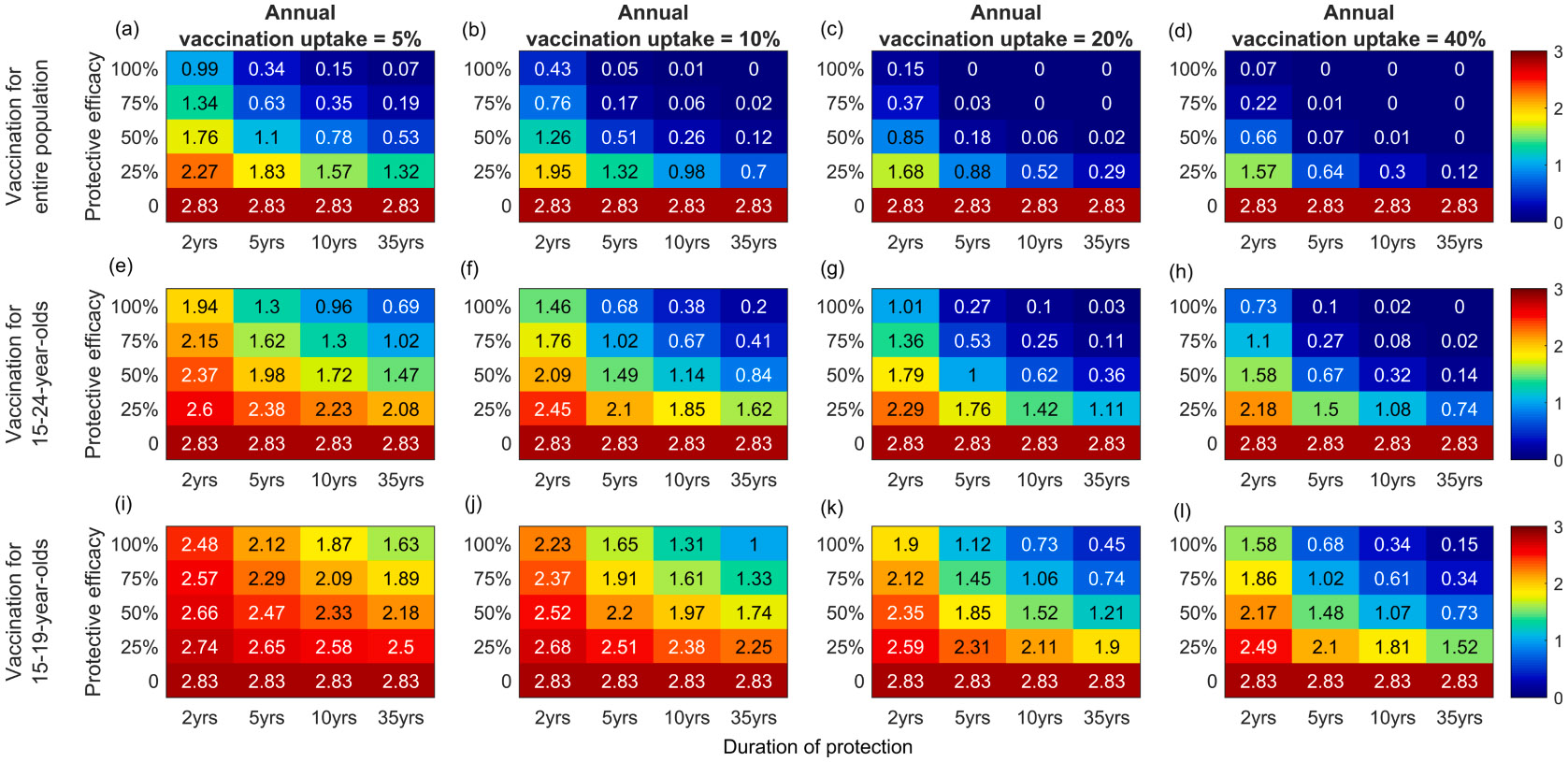
*N. gonorrhoeae* prevalence (percentage) in the entire population at 10 years following the introduction of vaccination. Vaccination is provided to: the entire population (top row); 15–24-year-olds (middle row); or 15–19-year-olds (bottom row). Vaccination confers protection against acquisition of infection only (Table 1, Scenario 1), with varying efficacy (0, 25 %, 50 %, 75 %, or 100 %) and duration of protection (2 years, 5 years, 10 years, or 35 years). Columns denote the annual vaccination uptake (5 %, 10 %, 20 % or 40 %). The heatmap colour key is shown on the right.

Vaccination of the entire population (15–49-year-olds; Fig. 3 a-d) has the greatest effect on *N. gonorrhoeae* prevalence 10 years after introduction of a vaccine that reduces susceptibility to infection, followed by more targeted 15–24-year-old (Fig. 3 e-h) and 15–19-year-old (Fig. 3 i-l) age-group vaccination programmes. For example, for a vaccine with 5-year duration of protection and 50 % protective efficacy, in the 15–49-year-old vaccination programme the entire population prevalence is reduced by ~60, 80, 90 and 100 % in 10 years following introduction of the vaccine, for annual vaccination uptake of 5, 10, 20 and 40 %, respectively (Fig. 3 a-d). In the more targeted 15–24-year-old vaccination programme, the entire population prevalence is reduced by ~30, 50, 70 and 80 % in 10 years for vaccination uptake of 5, 10, 20 and 40 %, respectively (Fig. 3 e-h). For the same vaccine characteristics, target programmes, and annual vaccination uptakes, the age-groupspecific reductions in prevalence (Fig. S4) are similar to the reductions predicted for the entire population (Fig. 3) 10 years after the introduction of vaccination.

#### b) Vaccination for high-activity individuals

**Fig. 4.**
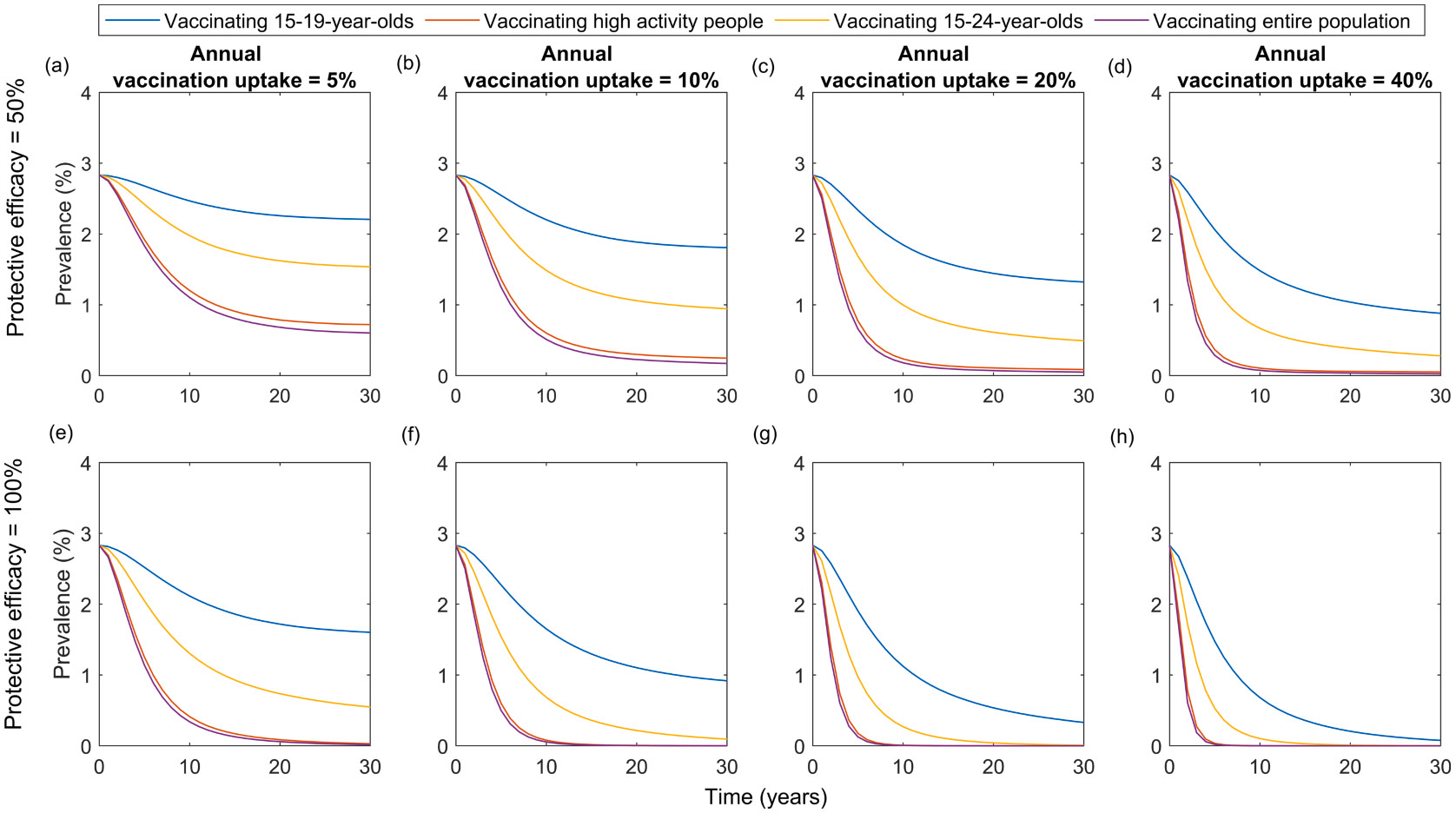
Change in prevalence over time in the entire population for different vaccination programmes (vaccinating the entire population, 15–24-year-olds, 15–19-year-olds or high-activity individuals) and annual vaccination uptakes (5 %, 10 %, 20 % or 40 %) if the vaccine is assumed to be 50 % (top row) or 100 % (bottom row) efficacious against acquisition of infection, with a duration of protection of 5 years.

In Fig. 4, we show the impact of vaccination over time for vaccination strategies targeting different age-groups as well as high-activity individuals. In the case of vaccines having 50 % or 100 % protective efficacy and 5-year duration of protection, programs targeting high-activity individuals are predicted to be almost as effective in reducing prevalence in the entire population as programs targeting the entire 15–49-year-old population for the same annual vaccination uptake in the target group. Reductions in prevalence are greater and more rapid in programmes targeting the high-activity group compared with those targeting the 15–19- and 15–24-year-old groups.

Results from our model suggest that vaccinating high-activity individuals is more efficient than vaccinating the entire population (Table 2). When considering 40 % annual vaccination uptake, the cumulative number of vaccinations needed to reduce prevalence in the entire population by 50 % or 90 % is ~3 times higher for the entire population programme than for the high-activity programme, although the time taken to achieve these reductions is similar in both programmes (~2 years and ~5 years to achieve 50 % or 90 % reduction, respectively). Vaccinating 15–24-year-olds is less efficient as it takes much longer to achieve a similar reduction in prevalence (~4 years and ~30 years to achieve 50 % and 90 % reduction, respectively). Similarly, when considering lower annual vaccination uptake of 5 %, 10 % or 20 %, the cumulative number of vaccinations needed to reduce prevalence in the entire population by 50 % or 90 % is consistently ~3 times higher for the entire population programme than for the high-activity programme. Here, it should be noted that ~35 % and ~30 % of the entire population are in the high-activity and 15–24-year-old group, respectively.

### 3.2. Vaccines that alter susceptibility to infection and/or transmissibility of infection, with 0–100 % protective efficacy and/or 0–100 % transmission suppression efficacy

**Fig. 5.**
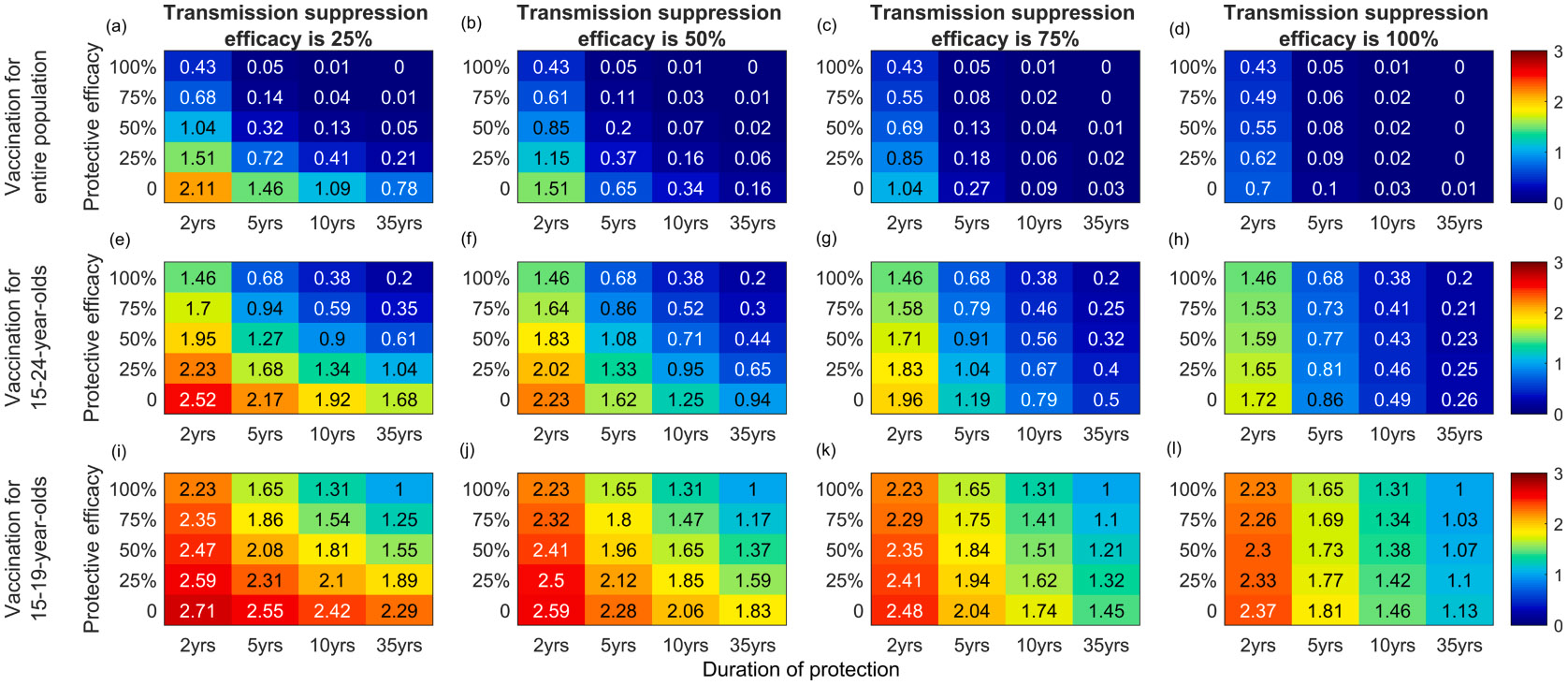
*N. gonorrhoeae* prevalence (percentage) in the entire population at 10 years following the introduction of vaccination when the annual vaccination uptake is 10 %. Vaccination is provided to: the entire population (top row); 15–24-year-olds (middle row); or 15–19-year-olds (bottom row). Vaccination confers protection against acquisition of infection and onward transmission (Table 1, Scenario 2), with varying protective efficacy (0, 25 %, 50 %, 75 %, or 100 %) and duration of protection (2 years, 5 years, 10 years, or 35 years). Columns denote the transmission suppression efficacy (25 %, 50 %, 75 % or 100 %). The heatmap colour key is shown on the right.

In Fig. 5, the additional effect on *N. gonorrhoeae* prevalence of a vaccine that reduces both susceptibility to infection and onward transmission by vaccinated individuals with a breakthrough infection is illustrated at an assumed annual vaccination uptake of 10 %. In general, for vaccines with <100 % protective efficacy the reduction in prevalence is predicted to be higher if the vaccine also reduces onward transmission with ≥ 25 % transmission suppression efficacy. For example, for a vaccination program in 15–24-year-olds with 5-year duration of protection, an additional 20 % reduction in prevalence is achieved with a vaccine that reduces susceptibility and transmissibility by 25 % (Fig. 5e) in comparison to one that only reduces susceptibility by 25 % (Fig. 3f). The impact of a vaccine that reduces transmissibility only is shown in Fig. 5 (last row of each heat map). If the annual vaccination uptake is 10 % and duration of protection is 5 years, the entire population prevalence is reduced by ~50, 80, 90 and 100 % in 10 years following introduction of the vaccine, when transmission suppression efficacy is 25, 50, 75 and 100 %, respectively in the entire population program.

### 3.3. Vaccines that alter susceptibility to infection, transmissibility of infection and probability of developing symptoms

**Fig. 6.**
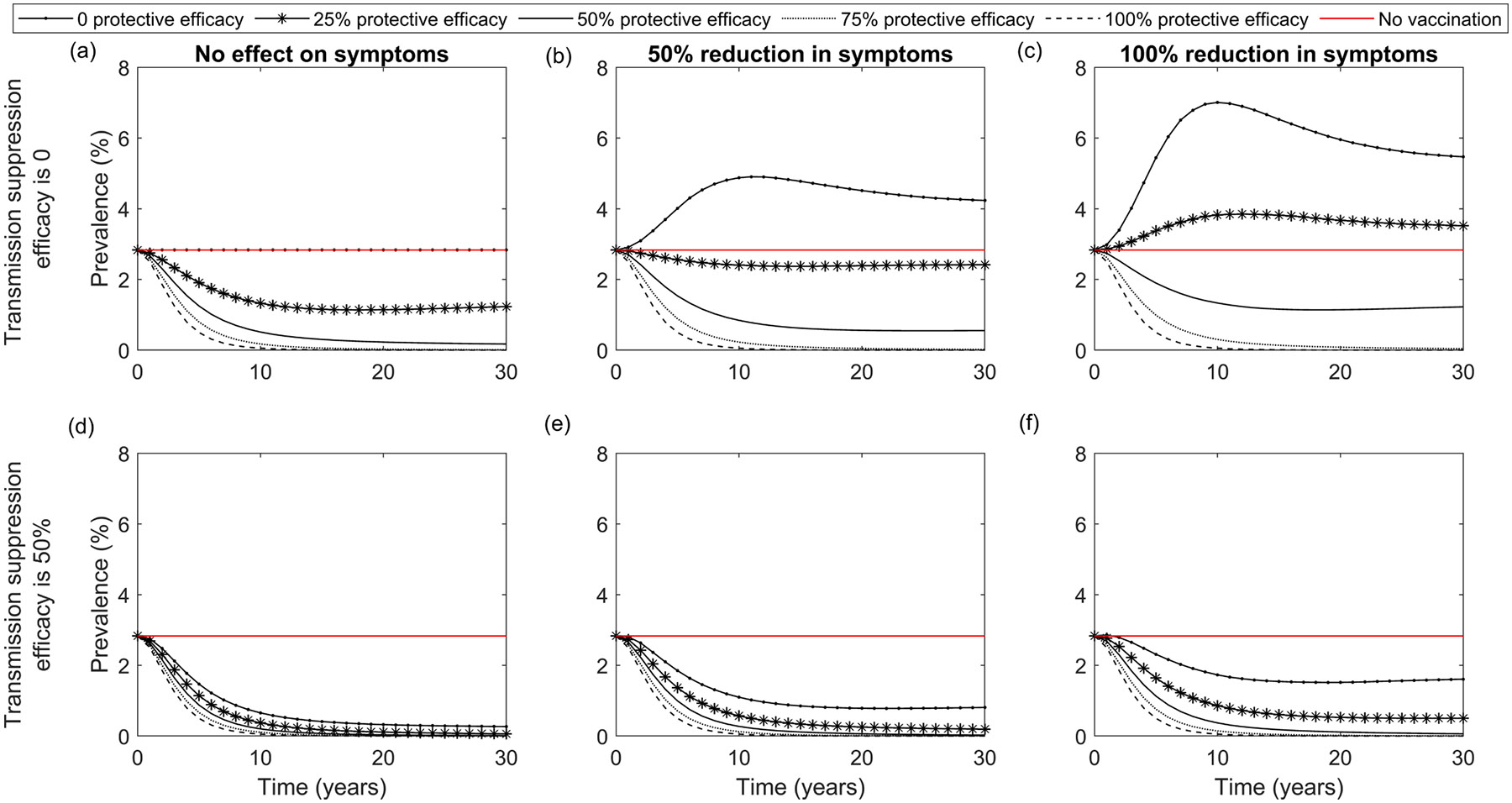
Prevalence over time in the entire population when vaccination has no effect on symptoms (left column), vaccination reduces the probability of development of gonorrhoea symptoms by 50 % (middle column) or by 100 % (right column). Top row: transmission suppression efficacy is 0 (1st row). Bottom row: transmission suppression efficacy is 50 % for varying protective efficacies (0 %, 25 %, 50 %, 75 %, 100 % as per legend). Vaccination is provided to the entire population, the duration of vaccine conferred protection is 5 years, and annual vaccination uptake is 10 %.

The impact of vaccines that prevent the development of symptoms after infection but do not provide complete protection against infection and transmission was also investigated, under the assumption that asymptomatic individuals will not seek treatment. The change in *N. gonorrhoeae* prevalence over time when vaccination reduces the probability of developing symptoms with 0–100 % protective efficacy and/or 0–50 % transmission suppression efficacy is shown in Fig. 6. In general, if the vaccine reduces the probability of developing gonorrhoea symptoms, reductions in prevalence will be less than when the vaccine reduces susceptibility only, under equivalent conditions (coverage, duration of protection). Indeed, *N. gonorrhoeae* prevalence is predicted to rise if the vaccine does not reduce susceptibility when symptoms are 50 % less likely to occur and to rise even with a 25 % reduction in susceptibility when symptoms do not occur in vaccinated people. However, when the vaccine reduces both susceptibility and transmissibility, the model predicts a noticeable reduction in *N. gonorrhoeae* prevalence even if the vaccine reduces the probability of symptoms.

## 4. Discussion

Interest in developing gonorrhoea vaccines is growing due to the increasing incidence of *N. gonorrhoeae* infections globally and the ongoing threat of antimicrobial resistance. The recently released WHO preferred product characteristics (PPC) for gonococcal vaccines aim to support and guide vaccine development and ensure that when a gonorrhoea vaccine becomes available it will have the optimal public health value, particularly for LMICs where the burden of *N. gonorrhoeae* infection is highest [10]. In this mathematical modelling study, we investigate the potential impact of a gonorrhoea vaccine in a setting with similar characteristics to a province in South Africa (KwaZulu-Natal), as an example of a LMIC setting with a high *N. gonorrhoeae* prevalence. Overall, we demonstrated that *N. gonorrhoeae* prevalence can potentially be substantially reduced by annual vaccination of only a proportion of the general heterosexual population with a vaccine that has partial efficacy against *N. gonorrhoeae* acquisition and/or transmission.

A vaccine that completely prevents infection is ideal, however this may not be achievable given the difficulties in developing a gonorrhoea vaccine to date [26], and future vaccines may only provide partial protection against infection, and/or reduce transmission. To identify desirable characteristics of a gonorrhoea vaccine for a high *N. gonorrhoeae* prevalence setting we considered three types of vaccine efficacy: efficacy against the acquisition of infection (*protective efficacy*); efficacy against transmission of infection *(transmission suppression efficacy)*; and efficacy in suppressing development of symptoms *(symptom suppression efficacy)*. According to our model, if protective efficacy is 25 % and duration of protection is 5 years, *N. gonorrhoeae* prevalence can be reduced by ~50 % in 10 years following introduction of a vaccine to the entire population with an annual vaccination uptake of only 10 %. A substantial decrease would still be seen with lower vaccine uptake. For example, a vaccine with 25–50 % protective efficacy and a 5-year duration protection can reduce *N. gonorrhoeae* prevalence by 35–60 % in the entire population within 10 years following the introduction of vaccination if 5 % of the entire population is vaccinated per year. These levels of vaccine efficacy are similar to findings from observational and case-control studies that have indicated that *N. meningitidis* serogroup B vaccines MeNZB [11] and 4CMenB [12,13] may provide cross protection against *N. gonorrhoeae*, with a predicted efficacy in the range 30–40 %.

The WHO PPC for gonococcal vaccines outlines preferred characteristics including 50 to 70 % efficacy or greater, and at least 10–15 years’ duration of protection for vaccinating young adolescents without a booster but highlights that a vaccine with 3–5 years duration could still provide benefits for older age-groups and specific high-risk populations [10]. Our study shows that the population implications of such a vaccine depend heavily on how that efficacy is manifested. If this efficacy is against acquisition of infection, our model indicates that even with 50 % efficacy and 5-year duration of protection, prevalence can be reduced by 82 % in 10 years following introduction of a vaccine to the entire population with an annual vaccination uptake of 10 %. If the vaccine is efficacious against infection acquisition as well as onward transmission (i.e., due to factors such as reduced bacterial load or duration of infection in vaccinated individuals with a breakthrough infection), the reductions in prevalence are even greater. Our model suggests that, if both the protective and transmission suppression efficacy of the vaccine is 50 %, prevalence can be reduced by 93 % in 10 years following introduction of a vaccine to the entire population with an annual vaccination uptake of 10 % and duration of protection of 5 years. However, if for example the vaccine only reduced onwards transmission, this effect could be missed in clinical trials unless specifically designed to measure the transmission suppression efficacy. Finally, for a vaccine that only reduces symptoms, population prevalence of infection can increase substantially despite high protection at an individual level against disease. These findings highlight the importance of understanding the mode of action of future vaccines.

We also considered vaccination programs targeted at different age- and sexual-activity groups, from the viewpoint of efficient use of resources. Globally, the highest incidence of *N. gonorrhoeae* infection typically occurs in the 15 to 24-year-old age group compared with older ages, although infections may also be high in older age-groups in populations at higher risk for infection [21,27-29]. According to our model, vaccinating a proportion of the entire 15–49-year-old population or a similar portion of high-activity individuals are the most effective vaccination programmes, and result in similar reductions in prevalence in the entire population. However, vaccinating only a proportion of the high-activity individuals is more efficient, in terms of the cumulative number of vaccines administered to achieve a given reduction in prevalence over a given time interval, compared to the other vaccination programmes that we considered. Although the younger age-group programmes (15–19- or 15–24-year-olds) are far less efficient compared to the other programs we evaluated, vaccinating the younger age-groups has the potential benefit of enabling vaccination before sexual debut, which may reduce the risk of early infections that go undetected/untreated and lead to sequelae, particularly in women of reproductive age.

The WHO target is to reduce the number of new *N. gonorrhoeae* cases among people 15–49 years old per year by 90 % from 2020 to 2030 [30]. While we do not present reductions in incidence here, our model predicts that *N. gonorrhoeae* prevalence can be reduced by ≥ 90 % in 10 years following introduction of the vaccine, if the vaccine has (i) ≥ 50 % protective efficacy, ≥10-year-duration of protection and ≥ 10 % of the entire unvaccinated population is vaccinated annually, or (ii) ≥ 75 % protective efficacy, ≥10-year-duration and if ≥ 20 % of 15–24-year-olds are vaccinated annually. If vaccination is provided to only 15–19-year-olds, to achieve a ~90 % reduction in 10 years, the vaccine needs to be 100 % protective against infection and the annual vaccination uptake should be 40 %. In South Africa and other LMIC settings, the availability of health infrastructure will play a key role in vaccine implementation strategies. The expansion of HIV testing and prevention programs may provide an opportunity for the inclusion of gonorrhoea vaccination. In South Africa, 45 % and 58 % of men and women, respectively, are tested for HIV annually [23], and the 5–40 % annual vaccine uptake that we modelled was based on scenarios where ~10–80 % of people who would annually be tested for HIV may receive vaccination against *N. gonorrhoeae*.

Other modelling studies have investigated the potential impact of gonorrhoea vaccines in heterosexual populations having *N. gonorrhoeae* prevalence of ~0.1–1.5 %, where vaccination was provided to young people prior to sexual debut [18-20]. Our study is the first to consider vaccine impact in a high prevalence (~2.8 %) heterosexual population in a LMIC setting. The study by Craig *et al.* [18], using a model that represents a sexually active general heterosexual population, predicted that a vaccine that is 20 % efficacious against infection/transmission could reduce *N. gonorrhoeae* prevalence by 40 % in 15–20 years under the optimistic assumptions that vaccine-conferred protection lasts for 20 years and coverage is 100 %. Vaccination was assumed to be administered at the age of 13 years prior to sexual debut and a specific geographical setting was not considered. Our model predicts that a similar reduction in prevalence could be achieved in about 10 years following the introduction of a vaccine with 25 % protective efficacy and 5 years duration of protection if annual vaccination uptake is 20 % in 15–24-year-olds (i.e., 13 % of the population are vaccine-protected). The modelling study by Carey *et al*. [19], that considered a heterosexual population living in the United States, found that 20 % population coverage, defined as the percentage of the population vaccinated prior to sexual debut, would lead to only a ~20 % reduction in *N. gonorrhoeae* prevalence in 10-years following introduction of vaccination with a vaccine of 30 % efficacy against infection that wanes after 5-years. The mathematical modelling study by Looker *et al.* [20] considered a heterosexual population in the United Kingdom and estimated that the percentage reduction in incident *N. gonorrhoeae* infections would only be 10 %, 18 % and 25 % in 10, 20 and 70 years, respectively, if the vaccine is 31 % efficacious against infection, wanes after 6 years by vaccinating 85 % of the 14-year-olds. The lower impact of vaccination predicted in these modelling studies is likely attributable to the different assumptions about sexual behaviour and age structure of the study populations. For example, Looker *et al.* [20] considered importation of infections from the MSM population which we did not include in our model, and the population sizes of the age-groups are unequal in their study (more older people than young people) while we considered age-groups of uniform size. Other studies do not stratify their models by age group; therefore, the partner change rates do not vary based on the age group as they do in our model. Another key difference is that we consider providing ongoing vaccination to specific age-groups annually rather than vaccination prior to sexual debut as implemented in the other modelling studies discussed here.

The relatively rapid reduction in *N. gonorrhoeae* prevalence following vaccination in our model is similar to other mathematical modelling studies that have noted that *N. gonorrhoeae* prevalence responds rapidly to interventions such as improved access to healthcare, condoms, and STI screening [31,32]. These modelling studies are consistent with the marked decline in *N. gonorrhoeae* incidence that was seen in high-income countries (e.g., the US) following the discovery of HIV, due to the increase in safe sex practices [33]. High sensitivity of prevalence to transmission-reducing interventions is expected when the basic reproduction number is only slightly above 1, supported for instance by a metapopulation model of*N. gonorrhoeae* transmission in the UK showing that even within high-risk groups, the reproduction number was < 1.2 [34]. Additionally, these larger effects on prevalence could also occur, because in our model, the proportion of people who are vaccine-protected is relatively high as vaccination is offered continuously to the target groups.

There are several limitations to our modelling study, which largely relate to the complexities of *N. gonorrhoeae* infection. Many distinct *N. gonorrhoeae* strains are likely to be circulating among heterosexuals at any given time [35], and vaccines may have differential efficacy depending on the strain. It is also possible that vaccine efficacy may differ for infection at different anatomical sites as is the case for antibiotic efficacy, which is typically lower for pharyngeal gonorrhoea infections compared to genital and rectal infections [36,37]. To reduce model complexity, we did not explicitly consider differential vaccination efficacy by *N. gonorrhoeae* strain or anatomical site, but rather consider an average efficacy across all strains and anatomical sites. While associations between bacterial load and transmissibility have been reported [38], bacterial load does not always correlate with symptoms or the severity of infection [39,40] and the link between symptoms and transmission is unclear. Our model therefore assumes that both asymptomatic and symptomatic infections are equally transmissible. Furthermore, we did not consider possible changes in sexual behaviour or treatment-seeking behaviour attributable to the introduction of the vaccine, such as the decreased risk-reduction behaviours (e.g., condom use) observed following PrEP introduction [41]. However, human papillomavirus vaccination has not been found to have had a noticeable effect on sexual behaviour among sexually active young adults in the US, Canada, and African settings [42-45]. In our model we assumed that people stay in the same activity class for their entire life. Strategies targeted at high-activity individuals might be less effective than predicted in our model if the high-activity individuals change their activity status frequently, as they may not be easily targeted for vaccination.

The model developed for this study is neither individual-based nor anatomically site-specific. An anatomical site-specific model would have allowed us to assess the impact of vaccines that differ in their ability to prevent infection at specific anatomical sites. While an individual based modelling approach would have facilitated this, the inclusion of anatomical sites requires information on many additional parameters, e.g., sexual act-specific transmission probabilities, duration of infection by anatomical site and the sex-specific proportion of urogenital, pharyngeal, and rectal infections that are symptomatic. Details and robust data to inform these parameters are unavailable and thus the additional complexity of such models introduces considerably greater uncertainty in the outcomes. We therefore made the pragmatic decision that a compartmental model was the most appropriate approach to address our research questions and to obtain informative results.

In conclusion, this modelling study provides some guidance on the vaccine characteristics and implementation strategies that could result in substantial gonorrhoea reductions in a high-prevalence setting. These findings will inform future vaccine development and implementation strategies and as well as vaccination programs that could help achieve WHO targets for reducing *N. gonorrhoeae* incidence by 90 % by 2030.

## Supplementary Material

1

Supplementary data to this article can be found online at https://doi.org/10.1016/j.vaccine.2023.07.048.

## Figures and Tables

**Table 1:** Vaccination scenarios and respective assumptions regarding duration of protection, annual vaccination uptake and efficacy. These scenarios are considered when providing vaccination for the entire population, 15–24-year-olds, 15–19-year-olds, or high-activity individuals.

Vaccine Scenario	Parameter value				
	Duration of vaccine-conferred protection (years)	Annual vaccination uptake (%)	Protective efficacy (%)	Transmission suppression efficacy (%)	Symptom suppression efficacy (%)*
1 **Vaccine reduces susceptibility to infection**	2/5/10/35^†^	5/10/20/40	0/25/50/75/100	0	0
2 **Vaccine reduces susceptibility to infection and/or transmissibility in vaccinated individuals**	2/5/10/35^†^	10	0/25/50/75/100	25/50/75/100	0
3 **Vaccine reduces susceptibility to infection and/or prevents the development of symptoms in a proportion of individuals with breakthrough infection**	5	10	0/25/50/75/100	0	50/100
4 **Vaccine reduces susceptibility to infection, transmissibility and/or prevents the development of symptoms in a proportion of vaccinated individuals with breakthrough infection**	5	10	0/25/50/75/100	0/50	50/100

* The probability of developing symptoms for vaccinated individuals who become infected is either 1, 0.5 or 0.

† Vaccinated individuals are protected for the entire 35-year modelled timespan, i.e., from entering to leaving the model population.

**Table 2:** Cumulative number of vaccines administered to achieve 50 % or 90 % reduction in *N. gonorrhoeae* prevalence in the entire population using different vaccination programmes. Vaccine numbers are shown for 40 % annual vaccination uptake in either the entire population, 15–24-year-olds, 15–19-year-olds, or high-activity individuals, with a vaccine that is 50 % efficacious against the acquisition of infection and has a 5-year duration of protection.

Vaccination programme	50 % reduction in prevalence		90 % reduction in prevalence	
	
	Time to achieve reduction (years)	Number of vaccinations per 1,000 population (IQR)	Time to achieve reduction (years)	Number of vaccinations per 1,000 population (IQR)
Entire population	1.9	639 (638–640)	5.1	982 (981–982)
15–24 only	4.3	296 (295–296)	30.0	982 (982–983)
15–19 only	10.9	300 (299–300)	>50	>1,000
High-activity individuals	2.1	244 (243–245)	5.7	358 (357–358)

## Data Availability

Model code will be made available upon request to the authors.
