# Supplementary material for: The potential impact of a vaccine on Neisseria gonorrhoeae prevalence among heterosexuals living in a high prevalence setting
Source: Vaccine. Author manuscript; Available in PMC 2025 Jun 30. (PMC12206584; doi:10.1016/j.vaccine.2023.07.048)
Supplement: 1 [file NIHMS2091667-supplement-1.pdf]

Supplementary data 1

Model description

A deterministic SIRS-type (Susceptible-Infected-Recovered-Susceptible) compartmental model [1] is used to describe *N. gonorrhoeae* transmission in heterosexuals aged 15-49 years. The natural history of *N. gonorrhoeae* is captured by allowing people to transition between four mutually exclusive states: susceptible ($S$); infected and seeking treatment due to symptoms ($I_s$; symptomatic and seeking treatment); infected and not seeking treatment ($I_{ns}$; primarily asymptomatic); and recovered ($R$; temporarily immune). The model tracks the flow of individuals between each state (compartment) over time. We assume that a high proportion (~0.7 for females and ~0.9 for males) of symptomatic individuals seek treatment (see Table S1). Those who do not seek treatment either recover naturally or are identified through testing and then treated. As treatment failure is rarely reported, we assume that treatment is always successful [2]. We also assume that infectiousness does not change with treatment, and we remove treated people directly to the recovered compartment. Following the resolution of infection, individuals remain in the recovered compartment for a short time (~7 days) before returning to the susceptible compartment since there is little evidence for enduring acquired immunity following resolution of *N. gonorrhoeae* infection [3].

Only heterosexual population is considered in the model, and the population is stratified by sex (female and male), sexual activity (high and low activity), and seven age groups (15-19, 20-24, 25-29, 30-34, 35-39, 40-44 and 45-49 years). Unless explicitly stated, natural history parameters are assumed to be the same across sex, age, and activity groups (defined in Table S1). We assumed that people remain in their assigned risk-group for the entire period they remain in the modelled population, since there is a lack of suitable data to inform the transition of people between high and low activity groups and. Our model is not anatomical-site-specific, hence parameters representing the duration of infection, the proportion of infections that are symptomatic and male-to-female and female-to-male transmission probabilities are considered to be an average over all anatomical routes of transmission.

Figure S1 shows relevant disease states and transitions of the model with the inclusion of gonorrhoea vaccination. Infection states for those who are vaccinated and protected or whose vaccine-conferred protection has waned are denoted by subscripts $V$ and $W$, respectively. As shown in Figure S1, unvaccinated individuals who receive vaccine move from the susceptible and recovered compartments of Panel A ($S$ and $R$ respectively) to the corresponding vaccinated compartments in Panel B ($S_V$ and $R_V$ respectively) at a rate $r_V = -log\,(1 - p_V)$, with $p_V$ denoted as the proportion of unvaccinated individuals who receive

vaccination in a year (refers to as annual vaccination uptake). We assume that all infected individuals who receive vaccine will receive treatment at the same time, and hence move to the vaccinated recovered compartment ($R_V$) following treatment, with this applied with rates $p_V r_{ns}$ and $p_V r_s$ for those not-seeking ($I_{ns}$) or seeking treatment ($I_s$) respectively. Here, $r_{ns}$ and $r_s$ refer to the recovery rates of people in $I_{ns}$ and $I_s$ compartments respectively. Vaccine protection wanes over time at a rate $\sigma$, and then vaccinated individuals (those in Panel B) move to the corresponding compartment in the vaccination waned state (Panel C).

The model is formulated as a system of ordinary differential equations (ODE) as follows:

$$\frac{dS_{kli}}{dt} = \omega R_{kli} - (\lambda_{kli} + r_V)\, S_{kli}$$

$$\frac{dI_{ns\,kli}}{dt} = (1 - p_k q_k)\lambda_{kli} S_{kli} - r_{ns} I_{ns\,kli}$$

$$\frac{dI_{s\,kli}}{dt} = p_k q_k \lambda_{kli} S_{kli} - r_s I_{s\,kli}$$

$$\frac{dR_{kli}}{dt} = (1 - p_V) r_{ns}\, I_{ns\,kli} + (1 - p_V) r_s I_{s\,kli} - (\omega + r_V) R_{kli}$$

$$\frac{dS_{V\,kli}}{dt} = \omega R_{V\,kli} - (\psi \lambda_{kli} + \sigma) S_{V\,kli} + r_V S_{kli}$$

$$\frac{dI_{Vns\,kli}}{dt} = (1 - \chi p_k q_k) \psi \lambda_{kli} S_{V\,kli} - (r_{ns} + \sigma) I_{Vns\,kli}$$

$$\frac{dI_{Vs\,kli}}{dt} = \chi p_k q_k \psi \lambda_{F,T} S_{V\,kli} - (r_s + \sigma) I_{Vs\,kli}$$

$$\frac{dR_{Vklj}}{dt} = r_{ns} I_{Vns\,kli} + r_s I_{Vs\,kli} + r_V\, R_{kli} + p_V r_{ns}\, I_{ns\,kli} + p_V r_s I_{s\,kli} - (\omega + \sigma) R_{V\,kli}$$

$$\frac{dS_{W\,kli}}{dt} = \omega R_{W\,kli} - \lambda_{kli} S_{W\,kli} + \sigma S_{V\,kli}$$

$$\frac{dI_{Wns\,kli}}{dt} = (1 - p_k q_k) \lambda_{kli} S_{Vkli} - r_{ns}\, I_{Wns\,kli} + \sigma I_{Vns\,kli}$$

$$\frac{dI_{Ws\,kli}}{dt} = p_k q_k \lambda_{kli} k S_{W\,kli} - r_s I_{Ws\,kli} + \sigma I_{Vs\,kli}$$

$$\frac{dR_{W\,klji}}{dt} = r_{ns}\, I_{Wns\,kli} + r_s I_{Ws\,kli} - \omega R_{W\,kli} + \sigma R_{V\,kli} \qquad \text{Eq. 1}$$

Here $k$ refers to gender, $l$ refers to sexual activity group and $i$ refers to age group ($i = 1$ youngest age group and $i = 7$ oldest age group).

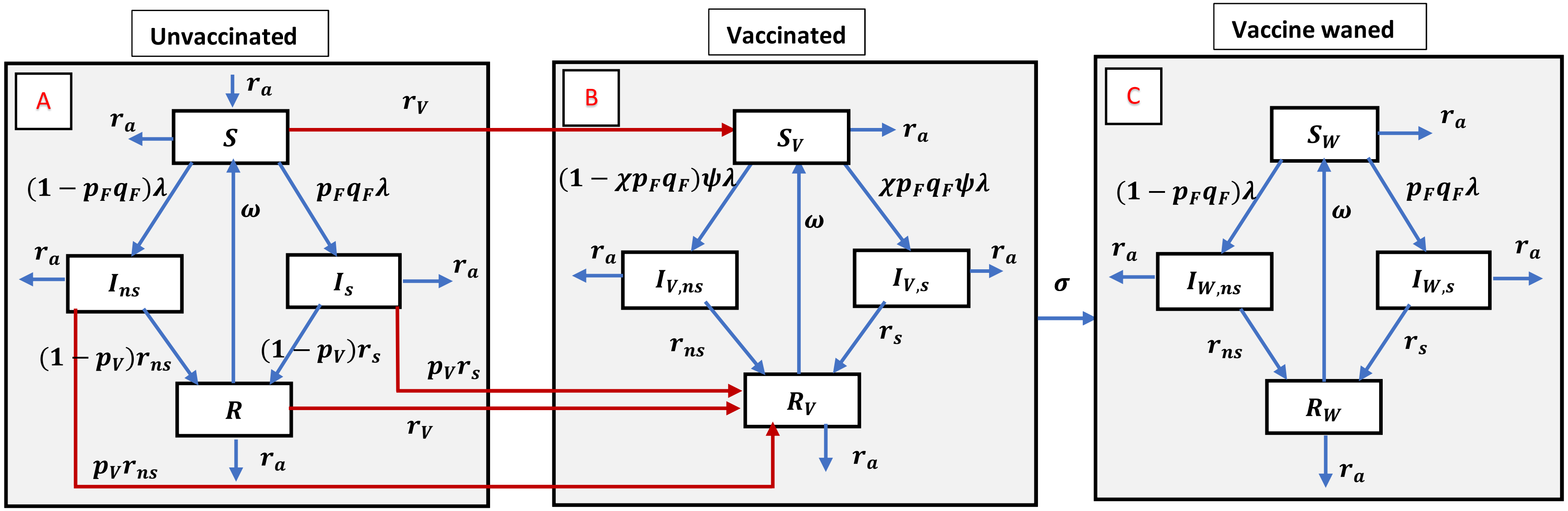


**Figure S1:** Schematic diagram of the model with vaccination. Panel A represents individuals who are unvaccinated, Panel B represents individuals who are vaccinated, and Panel C represent individuals whose vaccine-conferred protection has waned. Red arrows denote the transitions between unvaccinated and vaccinated model states and blue arrows denote all the other transitions.

Our model population is stratified into 5-year age groups, so that on average one fifth of people in each compartment move to the corresponding compartment in next older age group each year. Thus, the rate of aging, $r_a = \frac{1}{5} years^{-1}$. We assume that the total population size remains constant over time, so that the number of people leaving the oldest age group equals the number of people entering the youngest age group. We also assume that all new entries to the youngest age group are susceptible. The effect of aging is modelled as below.

$$\frac{dK_{kli}}{dt} = F(K_{kli}) - r_a K_{kli} + \begin{cases} r_a N_{kl7} & i = 1, K = S \\ 0 & i = 1, K \neq S \\ r_a K_{kl(i-1)} & i \neq 1 \end{cases}$$

$$N_{kli} = S_{kli} + I_{ns\,kli} + I_{s\,kli} + R_{kli} + S_{V\,kli} + I_{V\,ns\,kli} + I_{V\,s\,kli} + R_{V\,kli} + S_{W\,kli} + I_{W\,ns\,kli} + I_{W\,s\,kli} + R_{W\,kli}. \qquad \text{Eq. 2}$$

Here, $F(K_{kli})$ refers to the ODE for compartment $K$ as defined in Equation 1.

Parameter definitions and their assigned values (base case) are given in Table S1, with additional details/formulations for a subset of parameters provided below. The model was coded and implemented in MATLAB software (The Mathworks, Inc., Natick, MA). Model code will be made available upon request to the authors.

**Table S1:** Parameter descriptions and their assigned values (base case).

| Symbol | Description | Value* | Source |
|---|---|---|---|
| $p_k$ | Sex-specific proportion of infections that are symptomatic | F: 0.1-0.5<br>M: 0.3-0.7 | [4-7] |
| $q_k$ | Proportion of those with symptomatic infection who seek treatment | F: 0.7<br>M: 0.9 | According to Fairley *et al*. [8], virtually all men and most women who are symptomatic seek treatment. We assumed 0.9 for males because some men may not seek care if symptoms are not severe [9]. For females, a lower value of 0.7 is used since female symptoms are more likely to be mild or non-specific [10]. |
| $p_{sk}$ | Proportion of males and females tested annually | F: 0.2<br>M: 0.2 | Explained in Section 2. |
| $\theta$ | Sensitivity of screening test | 0.95 | Based on reported data, sensitivity is >90% [11-13]. |
| $\gamma$ | Rate of resolution of untreated infection | $\frac{1}{180}\,days^{-1}$ | [14-16], |
| $r_s$ | Rate of recovery after treatment | $\frac{1}{7}\,days^{-1}$ | [17, 18] |
| $\omega$ | The rate at which recovered individuals become susceptible to re-infection | $\frac{1}{7}\,days^{-1}$ | Assumption based on limited acquired immunity, following the resolution of infection immediate reinfection is deemed to be unlikely [19, 20]. |
| $\varepsilon_1$ | Mixing between age | 0.5 | Assumption |
| $\varepsilon_2$ | Mixing between activity | 0.5 | |

| | | | |
|---|---|---|---|
| $\boldsymbol{\varepsilon_3}$ | Differing degrees of mismatch between the ages of sexual partners | 0.5 | |
| $\boldsymbol{\beta_k}$ | Transmission probability per sexual act | F to M: 0.1-0.2<br>M to F: 0.1-0.4 | [5, 14] |
| $\boldsymbol{e}$ | Efficacy of condoms per sexual act | 0.95 | Based on [21, 22], condom efficacy is >0.90. |
| $\boldsymbol{c_{k,li}}$ | Age and activity group-specific average number of partners per annum for males. | Values are given in Table S5 below. | Estimated by model calibration. Female rates were calculated using male rates in order to balance the partnerships, as explained in [23]. |
| $\boldsymbol{p_c}$ | The proportion of acts in which condoms are used | 0.30 | Estimated by model calibration. See Section 3 below. |
| $\boldsymbol{n}$ | The average number of sexual acts per week for females and males in high and low sexual activity groups | 1.2 | Estimated by model calibration. See section 3 below. |

*Here, F and M refer to females and males, and H and L refer to high and low activity groups.

## 1. Parameter estimation

Rate of recovery of those who do not seek treatment ($r_{ns\,k}$): For those who do not seek treatment (regardless of symptoms), the infection resolves either through testing and treatment or through natural recovery. The resulting rate of recovery is then the sum of the testing rate in that population subgroup and the rate of natural recovery ($\gamma$). For a given test sensitivity $\theta$ and an annual proportion tested $p_{sk}$ we can define the testing rate as $-log(1 - p_{sk}\theta)$. Thus,

$$r_{ns\,k} = -log(1 - p_{sk}\theta) + \gamma. \qquad \text{Eq. 3}$$

As reported in [24] screening rate for males and females aged 15-24 years old is ~0.20. Due to the limited data on this parameter, we assumed this rate is uniform across 15-49-year-olds. To maintain this rate in our model we assumed that $p_{sk} = 0.20$ which gives $-log(1 - p_{sk}\theta) = 0.21$, with test sensitivity ($\theta$) of 0.95.

Force of infection ($\lambda_{kli}$): The force of infection (FOI) denotes the rate at which susceptible individuals of sex $k$, activity group $l$ and age group $i$ become infected and is given by:

$$\lambda_{kli} = c_{kli}\beta_{kli} \sum_{j=1}^{n_1} \sum_{m=1}^{n_2} \rho_{klmij} \frac{(I_{nsk'mj} + I_{sk'mj})}{N_{k'mj}} \qquad \text{Eq. 4}$$

In particular, this formula depends on the age and activity group specific new partner acquisition rates for females and males ($c_{kli}$), the transmission probability per sexual partnership ($\beta_{kli}$) and the probability that someone of sex $k$, age group $i$ and sexual activity group $l$ forms a partnership with someone from age group $j$ and sexual activity group $m$ of the opposite sex ($\rho_{klmij}$). The opposite sex is denoted by $k'$. The process for estimating each of these parameters is described in detail below.

Sexual mixing between activity groups: Mixing between sexual activity groups is implemented according to the formulation described in Garnett *et al.* [23]. The mixing parameter enables mixing to be varied between fully assortative and fully proportionate. In fully assortative mixing, all partners of an individual come from the same activity and age group of the opposite sex [23]. In fully proportionate mixing, the proportion of total contacts formed by individuals of one sexual activity and age group with individuals of the opposite sex and belonging to a particular activity and age group is proportionate to the relative size (i.e., proportion of the population in that group) of the group contributing the partners [25]. We define the degree of assortative mixing between age group as $\varepsilon_1$ and the degree of

assortative mixing between sexual activity classes as $\varepsilon_2$. When $\varepsilon_i = 1$, where $i = 1,2$ mixing is fully proportionate and when $\varepsilon_i = 0$, mixing is fully assortative. When $\varepsilon_i$ is assigned a value between 0 and 1, mixing is partially assortative/proportionate. Another parameter $\varepsilon_3$ was defined to denote the degree to which men form sexual relationships with women younger than themselves. Then the probability that someone of sex $k$, age group $i$ and sexual activity group $l$ forms a partnership with someone from age group $j$ and sexual activity group $m$ of the opposite sex is given by:

$$\rho_{klmij} = \left(\varepsilon_1 \frac{\sum_{u=1}^{2} N_{k'uj}}{\sum_{u=1}^{2}\sum_{v=1}^{7} N_{k'uv}} + (1-\varepsilon_1)\delta_{ij}\right)\left(\varepsilon_2 \frac{N_{k'mj}}{\sum_{u=1}^{2} N_{k'uj}} + (1-\varepsilon_2)\delta_{lm}\right)$$

$$\text{if } i,j \leq 2, i \neq j \text{ and } i \neq j+2.$$

$$\rho_{klmij} = \left[\left(\varepsilon_1 \frac{\sum_{u=1}^{2} N_{k'uj}}{\sum_{u=1}^{2}\sum_{v=1}^{7} N_{k'uv}} + (1-\varepsilon_1)\delta_{ij}\right) + \varepsilon_3\left(\varepsilon_1 \frac{\sum_{u=1}^{2} N_{k'uj+2}}{\sum_{u=1}^{2}\sum_{v=1}^{7} N_{k'uv}} + (1-\varepsilon_1)\delta_{ij+2}\right)\right]$$

$$\times\left(\varepsilon_2 \frac{N_{k'mj}}{\sum_{u=1}^{2} N_{k'uj}} + (1-\varepsilon_2)\delta_{lm}\right) \qquad \text{if } i = j+2.$$

$$\rho_{klmij} = (1-\varepsilon_3)\left(\varepsilon_1 \frac{\sum_{u=1}^{2} N_{k'uj}}{\sum_{u=1}^{2}\sum_{v=1}^{7} N_{k'uv}} + (1-\varepsilon_1)\delta_{ij}\right)\left(\varepsilon_2 \frac{N_{k'mj}}{\sum_{u=1}^{2} N_{k'uj}} + (1-\varepsilon_2)\delta_{lm}\right)$$

$$\text{if } i,j > 2 \text{ and } i = j.$$

Eq. 5

When calculating the mixing matrix elements, the balance rule needs to be applied so that the number of partnerships formed by males in age class $i$ and sexual activity class $l$ with females in age class $j$ and sexual activity class $m$ must equal those formed by women in age class $j$ and sexual activity class $m$ with men in age class $i$ and sexual activity class $l$. In accordance with the formulation by Garnett *et al.*, [23] this can be defined as below.

$$c_{kli}\rho_{klmij}N_{kli} = c_{k'mj}\rho_{k'mlji}N_{k'mj} \qquad \text{Eq. 6}$$

The values of $\rho_{kmlji}$ summed over $m$ and $j$ must equal unity since the elements of the mixing matrix are probabilities. To maintain the balance rule defined in Equation 6, we define the female partner change rates in terms of male partner change rates as,

$$c_{k'mj} = \frac{\sum_{l=1}^{2}\sum_{i=1}^{7} c_{kli}\rho_{klmij}N_{kli}}{N_{k'mj}} \qquad \text{Eq. 7}$$

and

$$\rho_{k'mlji} = \frac{c_{kli}\rho_{klmij}N_{kli}}{c_{k'mj}N_{k'mj}}. \qquad \text{Eq. 8}$$

<u>Partner acquisition rates</u> ($c_{kli}$): There were very limited data on the age-, gender- and activity-group-specific partner acquisition rates for KwaZulu-Natal, South Africa, the setting

we are considering in our model. So, to estimate age and activity-group-specific partner change rates we obtained data from the study by Garnett *et al.* [23] (see Table S2). According to Garnett *et al.* these data were comparable to the sexual behavioural patterns observed in the settings, Sub-Saharan, East, and West Africa. The prevalence study by Kharsany *et al.*, [26], considered for our model calibration, was not used when estimating partner change rates, as it does not give data on age-, gender-, and activity-group-specific partner change rates but provides data on overall male and female partner change rates. However, we used the Kharsany *et al.*, [26] study, to specify ranges for sexual behaviour parameters when calibrating the model (see Section 3).

**Table S2:** Partner acquisition rates for different age and activity groups relative to the minimum rate. These data were obtained from Garnett *et al.* [23].

| Activity group | Proportion of population | | Relative partner acquisition rate* |
|---|---|---|---|
| | Male | Female | |
| 1 | 0.1 | 0.05 | 100 |
| 2 | 0.3 | 0.25 | 20 |
| 3 | 0.4 | 0.5 | 5 |
| 4 | 0.2 | 0.2 | 1 |

| **Age group** | **15-19** | **20-24** | **25-29** | **30-34** | **35-39** | **40-44** | **45-49** |
|---|---|---|---|---|---|---|---|
| **Relative partner acquisition rate*** | 2 | 4 | 6 | 8 | 5 | 3 | 1 |

*Partner change rate of the group relative to the lowest group. For example, if partner acquisition rate of the 45-49-year-olds in activity group 4 is $a$ then for 15-19-year-olds in activity group 3 it is $a \times 2 \times 5$ and for 15-19-year-olds in activity group 1 it is $a \times 2 \times 100$. After model calibration $a = 0.029$, thus for 15-19-year-olds in activity groups 1 and 3, the partner acquisition rate is 5.80 and 0.29, respectively.

According to Table S2, if the average partner acquisition rate of the male population is $C$ per year, then

$$C = [a(2+4+6+8+5+3+1) \times 0.2 + 5a(2+4+6+8+5+3+1) \times 0.4 + 20a(2+4+6+8+5+3+1) \times 0.3 + 100a(2+4+6+8+5+3+1) \times 0.1]/7$$

where and $a$ is the partner acquisition rate of the 45-49-year-old in activity group 4. Here, we assume that the population sizes of the age groups are same. Now, we get $a = \frac{7C}{527.8}$, which is

the actual partner acquisition rate of 45-49-year-olds in activity group 4. Using this we can calculate actual partner acquisition rates for all groups.

Since our model has only two activity groups, we combined activity group 1 and 2 of Table S2 to be high-activity and 3 and 4 to be low activity and calculated the age and activity group specific partner acquisition rates. For example, we can calculate the partner change rate for the 15-19-year males of low activity group using the partner acquisition rate for 45-49, relative partner acquisition rate for 15-19, relative partner acquisition rate for males in activity group 3 and 4 and proportion of male in activity group 3 and 4. i.e., $\frac{2a\times 1\times 0.2+2a\times 5\times 0.4}{0.6}$.

We varied $C$ when calibrating the model (see Section 3 below). To balance the sexual partnerships among each group we estimated the female partner acquisition rates based on male rates as explained in Equation 7 above. Partner acquisition rates obtained, after calibrating the model for both males and females, are given in Table S5.

<u>Per-partnership transmission probability</u> ($\beta_{kli}$): We modelled transmission at a partnership level based on the duration of partnerships, the frequency of sexual encounters and condom-use on a per-act basis. Since data on the number of sexual encounters within a partnership for each sex and activity group ($n_{p\,kli}$) are not available, we assumed that the weekly number of sexual encounters ($n$) is the same for each sex, age, and activity group (see Table S1). The duration of each partnership is the inverse of the partner change rate (i.e., partnership duration = $\frac{1}{c_{kli}}$, such that for example, if someone has 3 partners per year, the partnership duration is 1/3 years). Then, $n_{p\,kli} = 52n\frac{1}{c_{kli}}$.

Next, we can define $(1-(1-e)\beta_k)^{n_{p\,kli}p_c}$ as the probability per-partnership of not being infected for sexual encounters where condoms were used, while $(1-\beta_k)^{n_{p\,kli}(1-p_c)}$ is the probability per-partnership of not being infected for sexual encounters in which condoms were not used. Here, $\beta_k$ refers to the transmission probability per sexual act, $e$ refers to the efficacy of condoms and $p_c$ is the proportion of acts in which condoms are used. The value of $p_c$ is assumed to be the same for all activity and age groups due to the lack of data on this parameter. Then the probability of transmission of infection per partnership per unit time is given by,

$$\beta_{kli} = 1-(1-(1-e)\beta_k)^{n_{p\,kli}p_c}(1-\beta_k)^{n_{p\,kli}(1-p_c)} \qquad \text{Eq. 9}$$

It should be noted here that, as the risk per partnership becomes saturated after a relatively small number of sex acts (due to the relatively high per-act transmission probability), the risk of acquiring infection is primarily proportional to the partner change rate and is relatively insensitive to plausible variations in the weekly number of sex acts.

## 2. Model calibration

Before applying vaccination, the model was calibrated to the reported age-specific prevalence data for KwaZulu-Natal district in South Africa in 2015 (see Table S3). We varied the average partner acquisition rate of the entire population, proportion of acts in which condoms are used among partnerships and number of sexual acts per week when calibrating the model, since these parameters are not well defined in the past literature. Although we used partner change rates in Garnett *et al.* [23] for parametrizing the model due to a lack of more recent data on these sexual behaviour parameters, we take account of the reported average partner acquisition rate for the entire population, weekly number of sexual acts, and the condom-use data reported in a few recent studies [26-28] compared to Garnett *et al.,* and specified ranges accordingly. The ranges used for the average partner acquisition rate of the entire population, proportion of acts in which condoms are used and number of sexual acts per week were, 1-3 [23, 26], 0.2-0.4 [26, 27] and 0.7-1.7 [28], respectively. The least squares curve fitting method (minimises the least squares distance between the modelled prevalence at equilibrium and the observed values) was used for calibration (using MATLAB function *lsqcurvefit*) and the values obtained for the average partner acquisition rate per year, proportion of acts in which condoms are used and number of sexual acts per week were 2.2, 0.3 and 1.2, respectively.

**Table S3:** The reported 2015 age-specific prevalence data for the district KwaZulu-Natal district in South Africa from the study by Kharsany *et al*. [26].

| Age group | Male (Median, 95% CI) | Female (Median, 95% CI) |
|---|---|---|
| **15-19** | 1.0 (0.3-1.6) | 4.2 (2.7-5.7) |
| **20-24** | 2.9 (1.4-4.5) | 6.5 (4.9-8.2) |
| **25-29** | 2.8 (1.1-4.4) | 4.4 (2.4-6.3) |

| | | |
|---|---|---|
| **30-34** | 2.5 (0.4-4.7) | 3.2 (1.6-4.8) |
| **35-39** | 0.5 (0.0-1.1) | 1.5 (0.7-2.3) |
| **40-44** | 0.7 (0.0-1.5) | 2.2 (0.6-3.8) |
| **45-49** | 0 | 1.1 (0.3-1.8) |

Calibration resulted in the model predicted age-group-specific prevalence presented in Figure S2 and male and female prevalence presented in Table S4. Model produced prevalence is quite similar to observed data for females and young males (<35-year-olds). However, for 35 and older male groups the model predicted prevalence were markedly higher. We do not assume change in sexual activity due to aging (e.g., high-activity people may become low-activity as they age) because of the lack of data regarding these behaviours and this could be a reason for high prevalence in older people in our model. It should also be noted that the reported prevalence data were based on the urine samples and vaginal swabs [26], and do not represent an average prevalence over all three anatomical sites. Therefore, the model produced prevalence could be reasonable when considering an average over all anatomical sites.

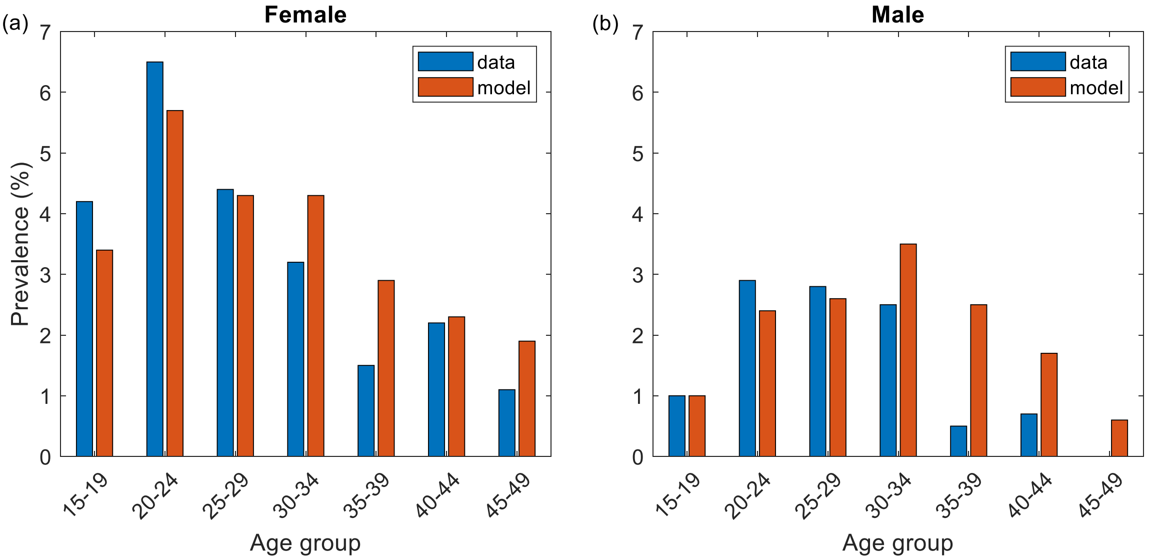


**Figure S2:** The model predicted (blue) and reported (red) age group specific prevalence for females and males.

**Table S4:** Comparing the model predicted and reported [26] prevalence data.

| Prevalence | Data (%) | Model (%) |
|---|---|---|
| **Female** | 3.7 (95% CI 3.1-4.3) | 3.6 |

| | | |
|---|---|---|
| **Male** | 1.8 (95% CI 1.0-2.5) | 2.0 |
| **Total (female and male)** | 2.8 (95% CI 2.3-3.3) | 2.8 |

With the obtained average partner acquisition rate of the entire population from the calibration, we can now estimate the age and activity group specific partner acquisition rates for males and females as discussed in the Section 2. These values are given in Table S5.

**Table S5:** Age and activity group specific partner change rates per annum for males and females. Female rates were obtained as described in Equation 7.

| Age group | Male | | Female | | Overall male | Overall female | Entire population |
|---|---|---|---|---|---|---|---|
| | High | Low | High | Low | | | |
| **15-19** | 2.33 | 0.21 | 4.99 | 1.49 | 1.06 | 2.54 | 1.80 |
| **20-24** | 4.67 | 0.43 | 6.63 | 1.98 | 2.13 | 3.38 | 2.76 |
| **25-29** | 7.00 | 0.64 | 4.99 | 1.49 | 3.18 | 2.54 | 2.86 |
| **30-34** | 9.34 | 0.86 | 4.85 | 1.45 | 4.25 | 2.47 | 3.36 |
| **35-39** | 5.84 | 0.53 | 3.58 | 1.07 | 2.65 | 1.82 | 2.24 |
| **40-44** | 3.50 | 0.32 | 2.83 | 0.85 | 1.59 | 1.44 | 1.52 |
| **45-49** | 1.17 | 0.11 | 2.39 | 0.71 | 0.53 | 1.21 | 0.87 |

Next, in order to allow for some flexibility around model predicted prevalence, we varied the natural history parameters, gender-specific per-act transmission probabilities and proportions of infections that are symptomatic. We generated 10,000 samples using Latin Hypercube Sampling (LHS) for these parameters (see the ranges used in Table S6) and the model was run for these 10,000 parameters sets until the equilibrium is reached. Then, at equilibrium, parameter sets which produced prevalence in the range 3.1%-4.3% for females and range 1.0%-2.5% for males (data on these ranges are shown in Table S4) were selected. While calibration to the age-specific data aimed at identifying optimal parameter choices for the three key sexual behaviour parameters, this latter simulation exercise aimed to refine plausible variation around per-act transmission probabilities and proportion of infections that are symptomatic. There were 572 samples that met the above criteria and the obtained median and Inter Quartile Ranges (IQR) for the varied parameters are given in Table S6 and their distributions in Figure S3.

**Table S6**: Parameter value ranges assigned to the natural history parameters that are varied and values obtained from selected 572 samples.

| Parameter* | Definition | Initial range | Obtained value (median; IQR) |
|---|---|---|---|
| $p_M$ | Proportion of infections that are symptomatic in men | 0.3-0.7 | 0.50 (0.42-0.56) |
| $p_F$ | Proportion of infections that are symptomatic in females | 0.1-0.5 | 0.25 (0.18-0.36) |
| $\beta_M$ | Female-to-male per-act transmission probability | 0.1-0.2 | 0.15 (0.13-0.18) |
| $\beta_F$ | Male-to-female per-act transmission probability | 0.1-0.4 | 0.28 (0.21-0.34) |

* The base case values used for these four parameters, when calibrating the model using least squares curve fitting method, were shown in Table S1.

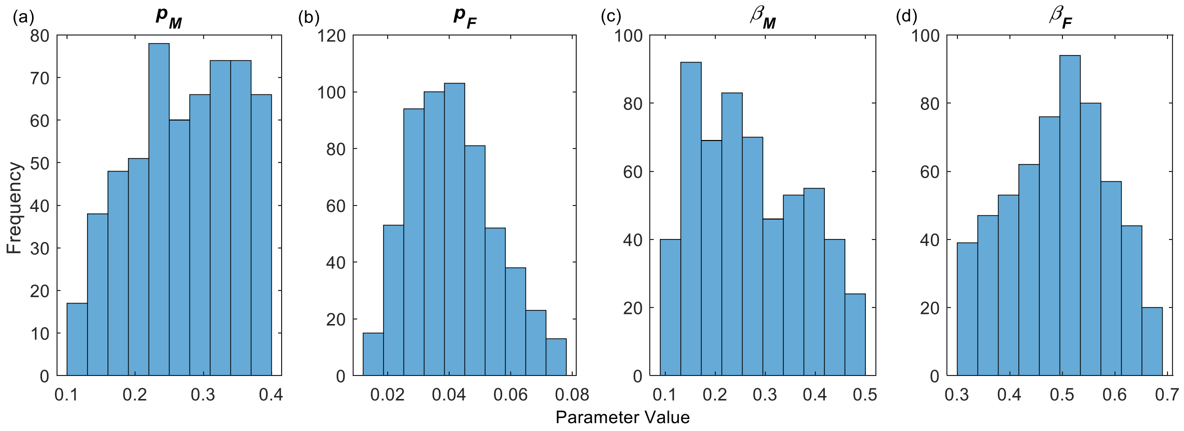


**Figure S3:** Distributions of parameter values of selected 572 samples.

## 3. Modelling typed of vaccine efficacies

After calibrating the model to 2015 prevalence data, we implemented vaccination scenarios from that time onwards. The characteristics of gonococcal vaccines investigated in our model are hypothetical since there are no vaccines available for *N. gonorrhoeae* infection at present. We considered three types of vaccine efficacies in this study and how they are modelled is discussed in the sections below.

a) Efficacy against infection (protective efficacy; $1 - \psi$):

If the vaccine efficacy against infection is $1 - \psi$, the FOI for vaccinated individuals reduces by a multiplicative factor of $\psi$, which is the relative susceptibility to infection of vaccinated individuals. So, if $\psi = 0$ the vaccine fully protects vaccinated individuals from becoming infected and when $\psi = 1$ the vaccine provides no protection from infection (see Panel B of Figure S1).

b) Efficacy against transmission (transmission suppression efficacy; $1 - \xi$):

If the parameter $\xi$, denotes the relative transmissibility of infection due to vaccination, then the vaccine efficacy against transmission is $1 - \xi$. To model this, we can adjust the FOI term as in Equation 10 below. Here too, if $\xi = 0$ the vaccine is 100% efficacious against transmission and if $\xi = 1$ the vaccine provides no protection against transmission.

$$\lambda_{kli} = c_{kli}\beta_{kli} \sum_{m=1}^{2} \sum_{j=1}^{7} \rho_{klmij} \left( \frac{I_{ns\,k'li} + I_{s\,k'li} + \xi(I_{Vns\,k'li} + I_{Vs\,k'li}) + I_{Wns\,k'li} + I_{Ws\,k'li}}{N_{k'li}} \right)$$

Eq. 10

c) Efficacy against the development of symptoms (symptom suppression efficacy; $1 - \chi$):

In this case, $\chi$ is the probability of a vaccinated individuals who become infected (see Panel B of Figure S1) to develop symptoms. If vaccination prevents the development of symptoms in vaccinated individuals who get infected, $\chi = 0$, and $\chi = 1$ if vaccine has no effect on symptoms.

## 4. Age-group specific *N. gonorrhoeae* prevalence

In Figure S4 below, we have presented the change in age group-specific prevalence for the two vaccination programmes, vaccination for entire population and vaccination for only 15-24-year-olds for different annual vaccination uptakes.

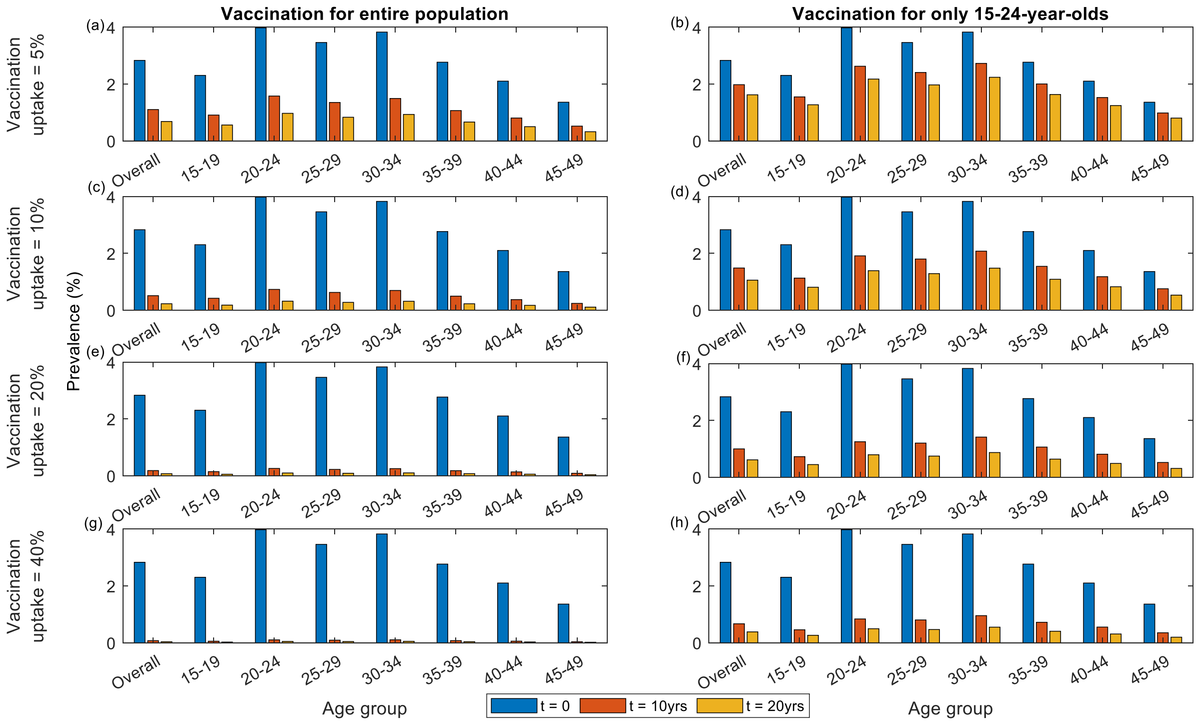


**Figure S4:** Age group specific *N. gonorrhoeae* prevalence at different time points ($t = 0$, $t = 10$ years and $t = 20$ years) for different annual vaccination uptakes (5%, 10%, 20% and 40%) and programmes (left column: vaccination for entire population and right column: vaccination for only 15-24-year-olds). Here, we assumed that the vaccine is 50% efficacious against infection (no efficacy against transmission or symptoms) and has a 5-year duration of protection.

## 5. Model-predicted median and interquartile ranges (IQR) for *N. gonorrhoeae* prevalence in the entire population

In the following tables we have presented both the median and IQR for *N. gonorrhoeae* prevalence in the entire population under the vaccination scenarios listed in Table 1 of the main text.

Vaccination reduces only the susceptibility to infection

**Table S7:** *N. gonorrhoeae* prevalence in the entire population at 10 years following introduction of vaccination to entire population if vaccination reduces only the susceptibility to infection.

| Annual vaccination uptake (%) | Protective efficacy (%) | Prevalence (%): Median (IQR) | | | |
|---|---|---|---|---|---|
| | | Duration of vaccine protection | | | |
| | | 2 years | 5 years | 10 years | 35 years |
| 5 | 100 | 0.99 (0.91-1.09) | 0.34 (0.31-0.37) | 0.15 (0.14-0.17) | 0.07 (0.06-0.07) |
| | 75 | 1.34 (1.23-1.46) | 0.63 (0.57-0.69) | 0.35 (0.32-0.39) | 0.19 (0.17-0.21) |
| | 50 | 1.76 (1.63-1.91) | 1.1 (1.01-1.2) | 0.78 (0.71-0.85) | 0.53 (0.48-0.58) |
| | 25 | 2.27 (2.10-2.44) | 1.83 (1.70-1.98) | 1.57 (1.45-1.70) | 1.32 (1.21-1.43) |
| | 0 | 2.83 (2.65-3.03) | 2.83 (2.65-3.03) | 2.83 (2.65-3.03) | 2.83 (2.65-3.03) |
| 10 | 100 | 0.43 (0.39-0.48) | 0.05 (0.05-0.06) | 0.01 (0.01-0.01) | 0 |
| | 75 | 0.76 (0.68-0.84) | 0.17 (0.16-0.19) | 0.06 (0.05-0.06) | 0.02 (0.02-0.02) |
| | 50 | 1.26 (1.15-1.38) | 0.51 (0.46-0.57) | 0.26 (0.23-0.28) | 0.12 (0.11-0.13) |
| | 25 | 1.95 (1.81-2.12) | 1.32 (1.22-1.45) | 0.98 (0.90-1.08) | 0.70 (0.64-0.77) |
| | 0 | 2.83 (2.65-3.03) | 2.83 (2.65-3.03) | 2.83 (2.65-3.03) | 2.83 (2.65-3.03) |
| 20 | 100 | 0.15 (0.13-0.17) | 0 (0-0.01) | 0 | 0 |
| | 75 | 0.37 (0.33-0.42) | 0.03 (0.03-0.03) | 0 (0-0.01) | 0 |
| | 50 | 0.85 (0.76-0.94) | 0.18 (0.16-0.20) | 0.06 (0.05-0.06) | 0.02 (0.01-0.02) |
| | 25 | 1.68 (1.54-1.83) | 0.88 (0.80-0.98) | 0.52 (0.47-0.58) | 0.29 (0.26-0.32) |
| | 0 | 2.83 (2.65-3.03) | 2.83 (2.65-3.03) | 2.83 (2.65-3.03) | 2.83 (2.65-3.03) |
| 40 | 100 | 0.07 (0.06-0.08) | 0 | 0 | 0 |
| | 75 | 0.22 (0.20-0.26) | 0.01 (0.01-0.01) | 0 | 0 |
| | 50 | 0.66 (0.58-0.74) | 0.07 (0.07-0.09) | 0.01 (0.01-0.01) | 0 |
| | 25 | 1.57 (1.43-1.73) | 0.64 (0.57-0.72) | 0.30 (0.26-0.33) | 0.12 (0.11-0.14) |
| | 0 | 2.83 (2.65-3.03) | 2.83 (2.65-3.03) | 2.83 (2.65-3.03) | 2.83 (2.65-3.03) |

**Table S8:** *N. gonorrhoeae* prevalence in the entire population at 10 years following introduction of vaccination to only 15-24-year-olds if vaccination reduces only the susceptibility to infection.

| Annual vaccination uptake (%) | Protective efficacy (%) | Prevalence (%): Median (IQR) | | | |
|---|---|---|---|---|---|
| | | Duration of vaccine protection | | | |
| | | 2 years | 5 years | 10 years | 35 years |
| 5 | 100 | 1.94 (1.80-2.10) | 1.30 (1.20-1.42) | 0.96 (0.88-1.04) | 0.69 (0.63-0.74) |
| | 75 | 2.15 (2.00-2.32) | 1.62 (1.49-1.75) | 1.30 (1.19-1.41) | 1.02 (0.93-1.10) |
| | 50 | 2.37 (2.21-2.55) | 1.98 (1.83-2.14) | 1.72 (1.59-1.86) | 1.47 (1.36-1.60) |
| | 25 | 2.6 (2.43-2.79) | 2.38 (2.22-2.56) | 2.23 (2.08-2.41) | 2.08 (1.93-2.24) |
| | 0 | 2.83 (2.65-3.03) | 2.83 (2.65-3.03) | 2.83 (2.65-3.03) | 2.83 (2.65-3.03) |
| 10 | 100 | 1.46 (1.34-1.59) | 0.68 (0.62-0.75) | 0.38 (0.34-0.42) | 0.20 (0.18-0.22) |
| | 75 | 1.76 (1.62-1.91) | 1.02 (0.93-1.12) | 0.67 (0.61-0.73) | 0.41 (0.37-0.45) |
| | 50 | 2.09 (1.93-2.26) | 1.49 (1.36-1.62) | 1.14 (1.04-1.24) | 0.84 (0.77-0.92) |
| | 25 | 2.45 (2.28-2.63) | 2.10 (1.94-2.26) | 1.85 (1.71-2.01) | 1.62 (1.49-1.75) |
| | 0 | 2.83 (2.65-3.03) | 2.83 (2.65-3.03) | 2.83 (2.65-3.03) | 2.83 (2.65-3.03) |
| 20 | 100 | 1.01 (0.91-1.11) | 0.27 (0.24-0.30) | 0.10 (0.09-0.11) | 0.03 (0.03-0.04) |
| | 75 | 1.36 (1.24-1.49) | 0.53 (0.47-0.58) | 0.25 (0.22-0.27) | 0.11 (0.09-0.12) |
| | 50 | 1.79 (1.64-1.94) | 1.00 (0.90-1.10) | 0.62 (0.56-0.68) | 0.36 (0.32-0.40) |
| | 25 | 2.29 (2.12-2.46) | 1.76 (1.62-1.91) | 1.42 (1.30-1.55) | 1.11 (1.02-1.22) |
| | 0 | 2.83 (2.65-3.03) | 2.83 (2.65-3.03) | 2.83 (2.65-3.03) | 2.83 (2.65-3.03) |
| 40 | 100 | 0.73 (0.65-0.82) | 0.10 (0.09-0.12) | 0.02 (0.02-0.03) | 0 (0-0.01) |
| | 75 | 1.10 (0.99-1.21) | 0.27 (0.24-0.30) | 0.08 (0.07-0.09) | 0.02 (0.02-0.03) |
| | 50 | 1.58 (1.45-1.73) | 0.67 (0.60-0.74) | 0.32 (0.28-0.36) | 0.14 (0.12-0.15) |
| | 25 | 2.18 (2.01-2.35) | 1.50 (1.37-1.64) | 1.08 (0.98-1.19) | 0.74 (0.66-0.81) |
| | 0 | 2.83 (2.65-3.03) | 2.83 (2.65-3.03) | 2.83 (2.65-3.03) | 2.83 (2.65-3.03) |

**Table S9:** *N. gonorrhoeae* prevalence in the entire population at 10 years following introduction of vaccination to only 15-19-year-olds if vaccination reduces only the susceptibility to infection.

| Annual vaccination uptake (%) | Protective efficacy (%) | Prevalence (%): Median (IQR) | | | |
|---|---|---|---|---|---|
| | | Duration of vaccine protection | | | |
| | | 2 years | 5 years | 10 years | 35 years |
| 5 | 100 | 2.48 (2.30-2.65) | 2.12 (1.96-2.28) | 1.87 (1.73-2.02) | 1.63 (1.51-1.76) |
| | 75 | 2.57 (2.39-2.75) | 2.29 (2.13-2.46) | 2.09 (1.94-2.25) | 1.89 (1.76-2.04) |
| | 50 | 2.66 (2.48-2.84) | 2.47 (2.30-2.64) | 2.33 (2.17-2.50) | 2.18 (2.03-2.35) |
| | 25 | 2.74 (2.57-2.94) | 2.65 (2.47-2.84) | 2.58 (2.40-2.76) | 2.50 (2.33-2.68) |
| | 0 | 2.83 (2.65-3.03) | 2.83 (2.65-3.03) | 2.83 (2.65-3.03) | 2.83 (2.65-3.03) |
| 10 | 100 | 2.33 (2.06-2.39) | 1.65 (1.53-1.78) | 1.31 (1.20-1.41) | 1.00 (0.92-1.08) |
| | 75 | 2.37 (2.20-2.55) | 1.91 (1.77-2.06) | 1.61 (1.49-1.74) | 1.33 (1.22-1.44) |
| | 50 | 2.52 (2.35-2.71) | 2.20 (2.04-2.37) | 1.97 (1.83-2.13) | 1.74 (1.61-1.88) |
| | 25 | 2.68 (2.50-2.87) | 2.51 (2.34-2.69) | 2.38 (2.22-2.56) | 2.25 (2.09-2.41) |
| | 0 | 2.83 (2.65-3.03) | 2.83 (2.65-3.03) | 2.83 (2.65-3.03) | 2.83 (2.65-3.03) |
| 20 | 100 | 1.90 (1.76-2.05) | 1.12 (1.03-1.22) | 0.73 (0.66-0.80) | 0.45 (0.41-0.49) |
| | 75 | 2.12 (1.96-2.28) | 1.45 (1.34-1.57) | 1.06 (0.97-1.16) | 0.74 (0.68-0.81) |
| | 50 | 2.35 (2.18-2.52) | 1.85 (1.71-1.99) | 1.52 (1.40-1.64) | 1.21 (1.11-1.31) |
| | 25 | 2.59 (2.41-2.77) | 2.31 (2.14-2.48) | 2.11 (1.95-2.27) | 1.90 (1.75-2.05) |
| | 0 | 2.83 (2.65-3.03) | 2.83 (2.65-3.03) | 2.83 (2.65-3.03) | 2.83 (2.65-3.03) |
| 40 | 100 | 1.58 (1.46-1.72) | 0.68 (0.62-0.75) | 0.34 (0.30-0.37) | 0.15 (0.13-0.17) |
| | 75 | 1.86 (1.72-2.01) | 1.02 (0.93-1.11) | 0.61 (0.55-0.67) | 0.34 (0.30-0.37) |
| | 50 | 2.17 (2.01-2.33) | 1.48 (1.37-1.61) | 1.07 (0.98-1.17) | 0.73 (0.67-0.81) |
| | 25 | 2.49 (2.32-2.67) | 2.10 (1.94-2.26) | 1.81 (1.67-1.95) | 1.52 (1.40-1.65) |
| | 0 | 2.83 (2.65-3.03) | 2.83 (2.65-3.03) | 2.83 (2.65-3.03) | 2.83 (2.65-3.03) |

Vaccination reduces both the susceptibility to infection and transmissibility of infection

**Table S10:** *N. gonorrhoeae* prevalence in the entire population at 10 years following introduction of vaccination to 10% of the entire population annually if vaccination reduces the susceptibility to infection and transmissibility of infection.

| Transmission suppression efficacy (%) | Protective efficacy (%) | Prevalence (%): Median (IQR) | | | |
|---|---|---|---|---|---|
| | | Duration of vaccine protection | | | |
| | | 2 years | 5 years | 10 years | 35 years |
| **25** | **100** | 0.43 (0.39-0.48) | 0.05 (0.05-0.06) | 0.01 (0.01-0.01) | 0 |
| | **75** | 0.68 (0.61-0.75) | 0.14 (0.12-0.15) | 0.04 (0.04-0.05) | 0.01 (0.01-0.01) |
| | **50** | 1.04 (0.94-1.14) | 0.32 (0.29-0.36) | 0.13 (0.12-0.15) | 0.05 (0.05-0.06) |
| | **25** | 1.51 (1.39-1.65) | 0.72 (0.65-0.79) | 0.41 (0.37-0.45) | 0.21 (0.19-0.24) |
| | **0** | 2.11 (1.95-2.28) | 1.46 (1.34-1.59) | 1.09 (1.00-1.19) | 0.78 (0.71-0.86) |
| **50** | **100** | 0.43 (0.39-0.48) | 0.05 (0.05-0.06) | 0.01 (0.01-0.01) | 0 |
| | **75** | 0.61 (0.55-0.68) | 0.11 (0.10-0.12) | 0.03 (0.03-0.03) | 0.01 (0.01-0.01) |
| | **50** | 0.85 (0.77-0.93) | 0.20 (0.18-0.22) | 0.07 (0.06-0.08) | 0.02 (0.02-0.02) |
| | **25** | 1.15 (1.04-1.26) | 0.37 (0.33-0.41) | 0.16 (0.14-0.17) | 0.06 (0.05-0.07) |
| | **0** | 1.51 (1.39-1.65) | 0.65 (0.59-0.72) | 0.34 (0.30-0.37) | 0.16 (0.15-0.18) |
| **75** | **100** | 0.43 (0.39-0.48) | 0.05 (0.05-0.06) | 0.01 (0.01-0.01) | 0 |
| | **75** | 0.55 (0.49-0.61) | 0.08 (0.07-0.09) | 0.02 (0.02-0.02) | 0 (0-0.01) |
| | **50** | 0.69 (0.62-0.76) | 0.13 (0.11-0.14) | 0.04 (0.03-0.04) | 0.01 (0.01-0.01) |
| | **25** | 0.85 (0.77-0.94) | 0.18 (0.17-0.21) | 0.06 (0.05-0.06) | 0.02 (0.02-0.02) |
| | **0** | 1.04 (0.95-1.15) | 0.27 (0.24-0.30) | 0.09 (0.08-0.10) | 0.03 (0.03-0.03) |
| **100** | **100** | 0.43 (0.39-0.48) | 0.05 (0.05-0.06) | 0.01 (0.01-0.01) | 0 |
| | **75** | 0.49 (0.44-0.55) | 0.06 (0.06-0.07) | 0.02 (0.01-0.02) | 0 |
| | **50** | 0.55 (0.50-0.62) | 0.08 (0.07-0.09) | 0.02 (0.02-0.02) | 0 |
| | **25** | 0.62 (0.56-0.69) | 0.09 (0.08-0.10) | 0.02 (0.02-0.02) | 0 (0-0.01) |
| | **0** | 0.70 (0.63-0.78) | 0.10 (0.09-0.12) | 0.03 (0.02-0.03) | 0.01 (0.01-0.01) |

**Table S11:** *N. gonorrhoeae* prevalence in the entire population at 10 years following introduction of vaccination to 10% of the 15-24-year-olds annually if vaccination reduces the susceptibility to infection and transmissibility of infection.

| Transmission suppression efficacy (%) | Protective efficacy (%) | Prevalence (%): Median (IQR) | | | |
|---|---|---|---|---|---|
| | | Duration of vaccine protection | | | |
| | | 2 years | 5 years | 10 years | 35 years |
| 25 | 100 | 1.46 (1.34-1.59) | 0.68 (0.62-0.75) | 0.38 (0.34-0.42) | 0.20 (0.18-0.22) |
| | 75 | 1.70 (1.56-1.84) | 0.94 (0.85-1.03) | 0.59 (0.53-0.64) | 0.35 (0.32-0.38) |
| | 50 | 1.95 (1.80-2.12) | 1.27 (1.16-1.38) | 0.90 (0.82-0.98) | 0.61 (0.56-0.67) |
| | 25 | 2.23 (2.07-2.40) | 1.68 (1.54-1.82) | 1.34 (1.23-1.46)) | 1.04 (0.95-1.13) |
| | 0 | 2.52 (2.35-2.71) | 2.17 (2.01-2.34) | 1.92 (1.78-2.08) | 1.68 (1.55-1.82) |
| 50 | 100 | 1.46 (1.34-1.59) | 0.68 (0.62-0.75) | 0.38 (0.34-0.42) | 0.20 (0.18-0.22) |
| | 75 | 1.64 (1.50-1.78) | 0.86 (0.78-0.94) | 0.52 (0.47-0.57) | 0.30 (0.27-0.33) |
| | 50 | 1.83 (1.68-1.98) | 1.08 (0.98-1.17) | 0.71 (0.64-0.77) | 0.44 (0.40-0.48) |
| | 25 | 2.02 (1.87-2.19) | 1.33 (1.22-1.45) | 0.95 (0.87-1.04) | 0.65 (0.59-0.71) |
| | 0 | 2.23 (2.07-2.40) | 1.62 (1.49-1.76) | 1.25 (1.15-1.36) | 0.94 (0.85-1.02) |
| 75 | 100 | 1.46 (1.34-1.59) | 0.68 (0.62-0.75) | 0.38 (0.34-0.42) | 0.20 (0.18-0.22) |
| | 75 | 1.58 (1.45-1.72) | 0.79 (0.72-0.87) | 0.46 (0.41-0.50) | 0.25 (0.23-0.28) |
| | 50 | 1.71 (1.57-1.85) | 0.91 (0.83-1.00) | 0.56 (0.50-0.61) | 0.32 (0.29-0.35) |
| | 25 | 1.83 (1.69-1.99) | 1.04 (0.95-1.14)) | 0.67 (0.60-0.73) | 0.40 (0.36-0.44) |
| | 0 | 1.96 (1.81-2.12) | 1.19 (1.09-1.30) | 0.79 (0.72-0.86) | 0.50 (0.45-0.54) |
| 100 | 100 | 1.46 (1.34-1.59) | 0.68 (0.62-0.75) | 0.38 (0.34-0.42) | 0.20 (0.18-0.22) |
| | 75 | 1.53 (1.40-1.66) | 0.73 (0.66-0.79) | 0.41 (0.37-0.44) | 0.21 (0.19-0.24) |
| | 50 | 1.59 (1.46-1.73) | 0.77 (0.70-0.84) | 0.43 (0.39-0.47) | 0.23 (0.20-0.25) |
| | 25 | 1.65 (1.52-1.80) | 0.81 (0.74-0.89) | 0.46 (0.42-0.50) | 0.25 (0.22-0.27) |
| | 0 | 1.72 (1.58-1.87) | 0.86 (0.78-0.94) | 0.49 (0.44-0.54) | 0.26 (0.23-0.29) |

**Table S12:** *N. gonorrhoeae* prevalence in the entire population at 10 years following introduction of vaccination to 10% of the 15-19-year-olds if vaccination reduces the susceptibility to infection and transmissibility of infection.

| **Transmission suppression efficacy (%)** | **Protective efficacy (%)** | **Prevalence (%): Median (IQR)** | | | |
|---|---|---|---|---|---|
| | | **Duration of vaccine protection** | | | |
| | | **2 years** | **5 years** | **10 years** | **35 years** |
| **25** | **100** | 2.23 (2.06-2.39) | 1.65 (1.53-1.78) | 1.31 (1.20-1.41) | 1.00 (0.92-1.08) |
| | **75** | 2.35 (2.18-2.52) | 1.86 (1.72-2.00) | 1.54 (1.42-1.67) | 1.25 (1.15-1.35) |
| | **50** | 2.47 (2.29-2.64) | 2.08 (1.92-2.23) | 1.81 (1.67-1.95) | 1.55 (1.43-1.67) |
| | **25** | 2.59 (2.41-2.77) | 2.31 (2.14-2.48) | 2.10 (1.95-2.26) | 1.89 (1.75-2.04) |
| | **0** | 2.71 (2.53-2.90) | 2.55 (2.37-2.73) | 2.42 (2.26-2.60) | 2.29 (2.13-2.46) |
| **50** | **100** | 2.23 (2.06-2.39) | 1.65 (1.53-1.78) | 1.31 (1.20-1.41) | 1.00 (0.92-1.08) |
| | **75** | 2.32 (2.15-2.49) | 1.80 (1.67-1.94) | 1.47 (1.36-1.59) | 1.17 (1.08-1.27) |
| | **50** | 2.41 (2.24-2.58) | 1.96 (1.81-2.11) | 1.65 (1.53-1.79) | 1.37 (1.26-1.48) |
| | **25** | 2.50 (2.32-2.68) | 2.12 (1.96-2.28) | 2.85 (1.71-2.00) | 1.59 (1.47-1.72) |
| | **0** | 2.59 (2.41-2.78) | 2.28 (2.12-2.45) | 2.06 (1.91-2.22) | 1.83 (1.69-1.97) |
| **75** | **100** | 2.23 (2.06-2.39) | 1.65 (1.53-1.78) | 1.31 (1.20-1.41) | 1.00 (0.92-1.08) |
| | **75** | 2.29 (1.12-2.46) | 1.75 (1.61-1.88) | 1.41 (1.30-1.52) | 1.10 (1.01-1.19) |
| | **50** | 2.35 (2.18-2.52) | 1.84 (1.70-1.99) | 1.51 (1.40-1.64) | 1.21 (1.11-1.31) |
| | **25** | 2.41 (2.24-2.59) | 1.94 (1.79-2.09) | 1.62 (1.50-1.75) | 1.32 (1.22-1.44) |
| | **0** | 2.48 (1.30-1.65) | 2.04 (1.89-2.19) | 1.74 (1.61-1.87) | 1.45 (1.33-1.57) |
| **100** | **100** | 2.23 (2.06-2.39) | 1.65 (1.53-1.78) | 1.31 (1.20-1.41) | 1.00 (0.92-1.08) |
| | **75** | 2.26 (2.10-2.43) | 1.69 (2.57-1.83) | 1.34 (1.24-1.46) | 1.03 (0.95-1.12) |
| | **50** | 2.30 (2.13-2.46) | 1.73 (1.60-1.87) | 1.38 (1.27-1.50) | 1.07 (0.98-1.16) |
| | **25** | 2.33 (2.16-2.50) | 1.77 (1.64-1.91) | 1.42 (1.31-1.54) | 1.10 (1.01-1.20) |
| | **0** | 2.37 (2.19-2.54) | 1.81 (1.68-1.96) | 1.46 (1.34-1.58) | 1.13 (1.04-1.23) |

Vaccination alter susceptibility to infection, transmissibility of infection and probability of developing symptoms

**Table S13:** *N. gonorrhoeae* prevalence in the entire population at 10 years following introduction of vaccination when vaccination reduces the probability of development of symptoms by 50% and by 100%. Here, 10% of the entire population is vaccinated annually and the duration of vaccine conferred protection is assumed to be 5 years.

| Transmission suppression efficacy (%) | Protective efficacy | Prevalence (%): Median (IQR) | | |
|---|---|---|---|---|
| | | No reduction in symptoms | 50% reduction in symptoms | 100% reduction in symptoms |
| **0** | **100** | 0.05 (0.05-0.06) | 0.05 (0.05-0.06) | 0.05 (0.05-0.06) |
| | **75** | 0.17 (0.16-0.19) | 0.23 (0.21-0.25) | 0.30 (0.27-0.34) |
| | **50** | 0.51 (0.46-0.57) | 0.84 (0.76-0.93) | 1.33 (1.14-1.51) |
| | **25** | 1.32 (1.22-1.45) | 2.41 (2.18-2.61) | 3.82 (3.28-4.31) |
| | **0** | 2.83 (2.65-3.03) | 4.88 (4.53-5.20) | 7.01 (6.31-7.60) |
| **50%** | **100** | 0.05 (0.05-0.06) | 0.05 (0.05-0.06) | 0.05 (0.05-0.06) |
| | **75** | 0.11 (0.10-0.12) | 0.12 (0.11-0.14) | 0.15 (0.13-0.16) |
| | **50** | 0.20 (0.18-0.22) | 0.27 (0.25-0.30) | 0.37 (0.33-0.42) |
| | **25** | 0.37 (0.33-0.41) | 0.57 (0.52-0.63) | 0.86 (0.75-0.97) |
| | **0** | 0.65 (0.59-0.72) | 1.10 (1.00-1.21) | 1.73 (1.49-1.95) |

## 6. References


1. Keeling, M.J. and P. Rohani, *Modeling infectious diseases in humans and animals*. 2008: Princeton University Press.
2. Kidd, S. and K.A. Workowski, *Management of gonorrhea in adolescents and adults in the United States.* Clinical infectious diseases : an official publication of the Infectious Diseases Society of America, 2015. **61 Suppl 8**(Suppl 8): p. S785-S801 DOI: http://doi.org/10.1093/cid/civ731.
3. Kraus, S.J., W.J. Brown, and R.J. Arko, *Acquired and natural immunity to gonococcal infection in chimpanzees.* J Clin Invest, 1975. **55**(6): p. 1349-56 DOI: http://doi.org/10.1172/jci108054.
4. *World Health Organization. Prevalence and incidence of selected sexually transmitted infections*. 2005.
5. Johnson, L.F., L. Alkema, and R.E. Dorrington, *A Bayesian approach to uncertainty analysis of sexually transmitted infection models.* Sex Transm Infect, 2010. **86**(3): p. 169-74 DOI: http://doi.org/10.1136/sti.2009.037341.
6. Whittles, L.K., P.J. White, and X. Didelot, *A dynamic power-law sexual network model of gonorrhoea outbreaks.* PLOS Computational Biology, 2019. **15**(3): p. e1006748 DOI: https://doi.org/10.1371/journal.pcbi.1006748.
7. Yaesoubi, R., et al., *Adaptive guidelines for the treatment of gonorrhea to increase the effective life span of antibiotics among men who have sex with men in the United States: A mathematical modeling study.* PLoS Med, 2020. **17**(4): p. e1003077 DOI: http://doi.org/10.1371/journal.pmed.1003077.
8. Fairley, C.K., *Access to health care is critical for the control of Sexually Transmitted Infections (STIs)*, in *HIV Australia*. 2018: Australian federation of aids organisations.
9. Farley, T.A., D.A. Cohen, and W. Elkins, *Asymptomatic sexually transmitted diseases: the case for screening.* Prev Med, 2003. **36**(4): p. 502-9 DOI: http://doi.org/10.1016/s0091-7435(02)00058-0.
10. McCormack, M., et al., *Clinical spectrum of gonococcal infection in women.* The Lancet, 1977. **309**(8023): p. 1182-1185 DOI: https://doi.org/10.1016/S0140-6736(77)92720-9.
11. Robert L. C., et al., *Systematic review: noninvasive testing for Chlamydia trachomatis and Neisseria gonorrhoeae.* Annals of internal medicine, 2005. **142**(11).

12. Crotchfelt, K.A., et al., *Detection of Neisseria gonorrhoeae and Chlamydia trachomatis in genitourinary specimens from men and women by a coamplification PCR assay.* Journal of clinical microbiology, 1997. **35**(6): p. 1536-1540 DOI: http://doi.org/10.1128/jcm.35.6.1536-1540.1997.
13. Gaydos, C.A., et al., *Comparison of three nucleic acid amplification tests for detection of Chlamydia trachomatis in urine specimens.* Journal of clinical microbiology, 2004. **42**(7): p. 3041-3045 DOI: http://doi.org/10.1128/JCM.42.7.3041-3045.2004.
14. Hui, B.B., et al., *The potential impact of new generation molecular point-of-care tests on gonorrhoea and chlamydia in a setting of high endemic prevalence.* Sex Health, 2013. **10**(4): p. 348-56 DOI: http://doi.org/10.1071/SH13026.
15. *World Health Organization. Prevalence and incidence: Chlamydia trachomatis, Neisseria gonorrhoeae, syphilis and Trichomonas vaginalis. Methods and results used by WHO to generate 2005 estimates.* 2011.
16. Garnett, G.P., et al., *The transmission dynamics of gonorrhoea: modelling the reported behaviour of infected patients from Newark, New Jersey.* Philos Trans R Soc Lond B Biol Sci, 1999. **354**(1384): p. 787-97 DOI: http://doi.org/10.1098/rstb.1999.0431.
17. *Australian STI Management Guidelines*. June 2019.
18. Whittles, L.K., P.J. White, and X. Didelot, *Estimating the fitness cost and benefit of cefixime resistance in Neisseria gonorrhoeae to inform prescription policy: A modelling study.* PLOS Medicine, 2017. **14**(10): p. e1002416 DOI: http://doi.org/10.1371/journal.pmed.1002416.
19. Plummer, F.A., et al., *Epidemiologic evidence for the development of serovar-specific immunity after gonococcal infection.* The Journal of Clinical Investigation, 1989. **83**(5): p. 1472-1476 DOI: http://doi.org/10.1172/JCI114040.
20. Arko, R.J., *An immunologic model in laboratory animals for the study of Neisseria gonorrhoeae.* The Journal of Infectious Diseases, 1974. **129**(4): p. 451-455 DOI: http://doi.org/10.1093/infdis/129.4.451.
21. Pinkerton, S.D. and P.R. Abramson, *Effectiveness of condoms in preventing HIV transmission.* Social Science & Medicine, 1997. **44**(9): p. 1303-1312 DOI: https://doi.org/10.1016/S0277-9536(96)00258-4.

22. Marfatia, Y.S., I. Pandya, and K. Mehta, *Condoms: Past, present, and future.* Indian journal of sexually transmitted diseases and AIDS, 2015. **36**(2): p. 133-139 DOI: http://doi.org/10.4103/0253-7184.167135.
23. Garnett, G.P. and R.M. Anderson, *Factors Controlling the Spread of HIV in Heterosexual Communities in Developing Countries: Patterns of Mixing between Different Age and Sexual Activity Classes.* Philosophical Transactions: Biological Sciences, 1993. **342**(1300): p. 137-159.
24. Esra, R.T. and L.F. Johnson, *Modelling the impact of screening for chlamydia and gonorrhoea in youth and other high-prevalence groups in a resource-limited setting.* International Journal of Public Health, 2020. **65**(4): p. 413-423 DOI: https://doi.org/10.1007/s00038-020-01351-0.
25. Blower, S.M., Mclean, A. R., *Mixing ecology and epidemiology.* Proc Biol Sci, 1991. **245**(1314): p. 187-92 DOI: http://doi.org/10.1098/rspb.1991.0108.
26. Kharsany, A.B.M., et al., *Population prevalence of sexually transmitted infections in a high HIV burden district in KwaZulu-Natal, South Africa: Implications for HIV epidemic control.* International Journal of Infectious Diseases, 2020. **98**: p. 130-137 DOI: https://doi.org/10.1016/j.ijid.2020.06.046.
27. Shisana O, et al., *South African National HIV Prevalence, Incidence and Behaviour Survey, 2012*. 2014, Cape Town: HSRC Press.
28. Johnson, L.F., et al., *Sexual behaviour patterns in South Africa and their association with the spread of HIV: Insights from a mathematical model.* Demographic Research, 2009. **21**: p. 289-340.
